# Development of an analytical method for accurate and precise determination of rare earth element concentrations in geological materials using an MC-ICP-MS and group separation

**DOI:** 10.3389/fchem.2022.906160

**Published:** 2023-01-12

**Authors:** Seung-Gu Lee, Kyung-Seok Ko

**Affiliations:** ^1^ Geology Division, Korea Institute of Geoscience and Mineral Resources, Daejeon, Republic of Korea; ^2^ Groundwater Environment Research Center, Korea Institute of Geoscience and Mineral Resources, Daejeon, Republic of Korea

**Keywords:** rare earth element, group separation, HIBA, MC-ICP-MS, geological materials

## Abstract

The concentration of rare earth elements (REEs) in geological materials including SLRS-6 (natural water certified reference material) and JB1b, JA1, and JG2 (Standard Rock Materials of Geological Survey of Japan) can be used as a tracer to characterize various geochemical processes in earth systems. Particularly, accurate and precise determination of rare earth element concentration in natural waters is difficult due to their extremely low concentration and the interference of polyatomic oxides. In this study, we developed a method for accurate and precise determination of the REE (particularly heavy rare earth elements) concentrations in geological materials including natural waters using a multi-collector inductively coupled plasma mass spectrometer (MC-ICP-MS) and group separation by 2-hydroxyisobutyric acid (HIBA). The REEs were separated into light rare earth elements (LREEs, La–Ce–Pr–Nd), middle rare earth elements (MREEs, Sm–Eu–Gd–Tb), and heavy rare earth elements (HREEs, Dy–Ho–Er–Tm–Yb–Lu) by a cation-exchange column (AG50W-X8 200–400 mesh) using HIBA. The recovery rates of each REE in the natural water sample exceeded 98%, whereas the recovery rates of each REE in rock materials exceeded 95% except for HREEs. The method developed in this study can accurately measure the REE concentrations (particularly HREE) in geological materials without polyatomic oxide interference during the REE analysis by using the MC-ICP-MS and, thus, can correctly interpret the geochemical implications of REEs in geological systems. The determination of the Sr concentrations and Sr isotopic ratios of SLRS-6 CRM and JB1b, JA1, and JG2 SRMs is also reported, and they are shown to be in good agreement with the recommended values.

## 1 Introduction

The rare earth elements (REEs) consist of 14 lanthanide elements (except Pm which has no natural isotope) ranging from La to Lu and have similar chemical and physical properties, with a gradual change in the ionic radius (the so-called lanthanide contraction). Such uniformity arises because of the similar configuration of the valence electrons in all the REEs and becomes a cause for an ordered distribution in various geological processes. Particularly, the REEs tend to exist in any natural occurrence as a group rather than as a single element or as a combination of a few of their numbers. They also have unique geochemical features during various geological processes such as chemical weathering and sedimentation. Therefore, the REE concentrations and their variation in natural waters can be used as a tracer to characterize various geochemical processes of water systems (Elderfield and Greaves, 1982; Dia et al., 2000; Lawrence et al., 2006; Lawrence and Kamber, 2006; Wu et al., 2007; Noack et al., 2014; Pignotti et al., 2017). However, the REE concentrations in natural waters are usually very low (generally, pg/mL level). Thus, accurate, precise, and reliable methods for their determination are a pre-requisite for interpreting the geochemical implications in environmental media such as river water, groundwater, and sea water. For example, Yeghicheyan et al. (2019) and Babechuk et al. (2020) reported compiled data for REE concentrations of SLRS-6 (natural river water certified reference material of NRC-CNRS). In the PAAS (Post-Archean Australian Shale)-normalized REE pattern, the shape of the HREE pattern shows some differences between these elements. In particular, the HREE pattern presented by Babechuk et al. (2020) indicated the possibility of a specific pattern called the REE tetrad effect (Peppard et al., 1969; Masuda and Ikeuchi, 1979; Masuda et al., 1987; Kawabe et al., 1998; Anenburg and Williams, 2022). However, the HREE pattern presented by Yeghicheyan et al. (2019) shows a zigzag shape rather than the REE tetrad effect. Such a difference can lead to different geochemical interpretations.

Inductively coupled plasma quadruple mass spectrometry (ICP-QMS) is the most popular and effective instrumental technique for the determination of the REE concentrations in geological, environmental, and meteoritic materials (Lawrence et al., 2006; Wu et al., 2007; Chung et al., 2009; Zhu et al., 2010; 2021; Tepe and Bau, 2015; Fischer and Kara, 2016; Pignotti et al., 2017; Tanaka et al., 2018; Babechuk et al., 2020; Wysocka, 2021). Recently, Babechuk et al. (2020) reported a method for the precise and accurate determination of the REE concentrations in natural river water using ICP-QMS. The authors mentioned that the Eu concentration showed a large discrepancy compared to most of the REEs due to the BaO^+^ interference. In fact, the Ba oxides can be problematic for the accurate and precise measurement of ^151^Eu^+^ and ^153^Eu^+^ due to ^135^Ba^16^O^+^ and ^137^Ba^16^O^+^ interference, respectively. Kim et al. (2019) experimentally showed that the Eu concentration increases with an increase in the Ba/Eu concentration ratio despite oxide correction during measurements of the REEs using the ICP-QMS technique. For example, the sample solution with a Ba concentration 100 times higher than the Eu concentration showed a measured value of the Eu concentration of 10% or higher than the recommended value. Since most natural waters have a Ba content 100 times higher than that of Eu, it may be necessary to remove Ba in order to determine the accurate and precise Eu concentration in natural river water because the concentration of Ba affects that of Eu in chondrite- or PAAS (Post-Archean Australian Shale)-normalized REE patterns.

The MC-ICP-MS also allows the measurement of multiple isotopes using techniques such as isotope dilution and standard sample bracketing to achieve more precise and accurate elemental concentrations (Baker et al., 2002; Kent et al., 2004; Pourmand et al., 2012). Compared to ICP-QMS, which has the disadvantage of being expensive, the MC-ICP-MS minimizes the influence of fluctuations inherent in the plasma source. However, for accurate and precise measurement of the REE concentration by using the MC-ICP-MS, the following conditions are required: 1) low procedural total blanks; 2) removal of the interfering matrix; and 3) elimination of molecular oxides and hydrides which result in direct interferences with the masses of the analytes. The determination of the REE concentration using ICP-MS can be distorted by spectral interferences that include the oxides of the lighter REEs on some of the heavier REEs such as ^140^Ce^16^O^+^ on ^156^Gd^+^, ^141^Pr^16^O^+^ on ^157^Gd^+^, and ^159^Tb^16^O^+^ on ^175^Lu^+^. In addition, the Ba oxides can also be problematic for the accurate and precise measurement of ^151^Eu^+^ and ^153^Eu^+^ due to ^135^Ba^16^O^+^ and ^137^Ba^16^O^+^ interference, respectively. Therefore, a preconcentration and a matrix separation step of the REEs is necessary for MC-ICP-MS analysis. The oxide interferences during measurement of the REEs by the MC-ICP-MS in this study are summarized in Table 1.

**TABLE 1:** REE isotopes used for determining concentrations by using the MC-ICP-MS and their isobaric interference isotopes. The contribution of the oxide interferences to the intensity signal of each REE group was assessed by measuring (L, M, H) REEO+/(L, M, H)REE ratios in 10 ng/mL multielement standard solutions *via* a Cetac Aridus II desolvating system (Wysocka, 2021).

	REE isotope	Isotopic abundance (%)	Interference oxide	Oxide and hydroxide rates produced by neighboring REE (MREE isobars by LREE oxides and HREE isobars by MREE oxide) (%)
LREE	^139^La	99.91119	^138^Ba1H+	-
	^140^Ce	88.449		-
	^141^Pr	100		-
	^143^Nd	12.173		-
	^144^Nd	23.973		-
	^145^Nd	8.293		-
MREE	^147^Sm	15	^130^Ba16O1H+	-
	^149^Sm	13.82	^132^Ba16O1H+	-
	^151^Eu	47.81	^135^Ba16O+, ^134^Ba16O1H+	-
	^153^Eu	52.19	^137^Ba16O+, ^136^Ba16O1H+	-
	^155^Gd	14.8	^139^La16O+, ^138^Ba16O1H+	0.183
	^157^Gd	15.65	^141^Pr16O+	0.028
	^159^Tb	100	^143^Nd16O+	0.024
HREE	^161^Dy	18.889	^145^Nd16O+, ^144^Sm17O+	0.024
	^163^Dy	24.896	^147^Sm16O+	0.019
	^165^Ho	100	^149^Sm16O+	0.047
	^167^Er	22.869	^151^Eu16O+	0.004
	^169^Tm	100	^153^Eu16O+	0.006
	^172^Yb	21.686	^156^Gd16O+, ^156^Dy16O+	0.029
	^173^Yb	16.103	^157^Gd16O+	0.019
	^175^Lu	97.401	^159^Tb16O+	0.011

In this article, we present the development of a modified method of MC-ICP-MS using group separation of the REEs by HIBA (2-hydroxyisobutric acid) to determine more accurately and precisely the concentration of the REEs in geological materials including natural waters. The analytical procedures, including their precision, accuracy, method blank, and the limits of quantification, are discussed. The REE concentration of the natural river water certified reference material (SLRS-6, NRC-CNRS) has been measured and compared with high-precision data measured by various ICP-MS techniques from the literature to examine the accuracy of our protocol. We also measured the REE concentrations of Standard Reference Materials (SRMs) such as JB1a, JA1, and JG2 produced by the Geological Survey of Japan (GSJ) for confirming the usefulness of the method developed in this study. When measuring the REE concentrations by using the MC-ICP-MS, our method almost completely eliminates the effect of polyatomic oxide interference, leading to a clearer understanding of the geochemical implications of HREEs in natural water.

## 2 Experimental procedures

### 2.1 Chemical reagents and samples

Ultrapure^®^ grade HNO_3_ (60%) and HCl (30%) used in this study were purchased from Merck (Darmstadt, Germany). Cation-exchange resin Biorad^®^ AG50W-X8 (200–400 mesh) and 2-hydroxyisobutric acid (HIBA, Tokyo Chemical Industry Co., LTD.) were used for the REE concentration and group separation. Single-element standard (STD) solutions (1 μg/g for atomic analysis) of the REEs were purchased from PerkinElmer, Inc. We also purchased single-element standard solutions (100 μg/g for atomic analysis) from AccuStandard for comparison with the PerkinElmer standard. All diluted solutions were prepared using deionized water from a Milli-Q system (Milli pore, Bedford, MA, United States) and stored in polypropylene bottles. A natural river water certified reference material (CRM SLRS-6) for the determination of the REE concentration in natural water was purchased from the National Research Council Canada. The Standard Reference Materials (SRMs) for rock samples such as JB1b, JA1, and JG2 were purchased from the Geological Survey of Japan (GSJ).

### 2.2 Sample digestion of rock samples, REE preconcentration, and ion exchange chromatography

Sample digestion procedures of the SRMs for rock samples followed an approach in Lee et al. (2016). Approximately 30–100 mg of each sample powder was dissolved in a 2:1 mixture of 2–4 mL of concentrated HF (29 M) and 1–2 mL of concentrated HNO_3_ (16 M) at ca. 160°C for more than 72 h in 15 mL Savillex vials. After the addition of 0.1–0.2 mL of concentrated HClO_4_, the dissolved sample solution was heated to dryness at ca. 180°C for more than 1 day. The cakes were re-dissolved in a mixture of 1 mL concentrated HCl and 0.5 mL concentrated HNO_3_ and then dried at ca. 160°C for 1 day. Sample residues were re-dissolved in 5–10 ml 6 M HCl stock solution. Of this, 0.5–2 ml was used to determine REE concentrations using the conventional inductively coupled plasma mass spectrometry (ICP-MS) method or column chromatography for REE preconcentration. The aliquots were dried and re-dissolved in 4 mL of 2 M HCl for HCl column chromatography.

For the natural water sample, although this method is not generally suitable because it frequently leads to precipitation of salts and is time-consuming and labor-intensive, we selected the simple method of evaporation for preconcentration and group separation of the REEs in water samples. Aliquots (50 mL) of SLRS-6 CRM water were placed in five pre-cleaned PTFE beakers for preconcentration, and then, the weight was measured. The water samples in the PTFE beakers were placed on a hot plate and evaporated slowly at 80°C. In order to prevent precipitation of salt, we stopped drying the water samples when less than 0.5 mL of the original aliquot remained in the PTFE beaker. Then, prior to sample loading for HCl column chromatography, 2 mL of 2N HCl was added to each PTFE beaker.

Tanaka et al. (2018) developed a group separation method for REE determination in GSJ/AIST geochemical references JCp-1 (coral) and JCt-1 (giant clam) using isotope dilution ICP-QMS (quadrupole mass spectrometry). The authors separated the REE fraction from major elements using Biorad AG50W X-8 resin (200–400 mesh) and HCl. The authors then divided the REEs into LREE (La, Ce, Pr, and Nd), MREE (Sm, Eu, Gd, Tb, and Dy), and HREE (Ho, Er, Tm, Yb, and Lu) fractions using a quartz glass column (ø 3 mm, length 98 mm) containing 0.8 mL of the cation-exchange resin (Biorad AG 50WX-8, 200–400 mesh) and 2-hydroxyisobutyric acid (HIBA) as the eluant. However, in the MREE by Tanaka et al. (2018), Dy (^161^Dy^+^) was masked by Sm oxides (^144^Sm^17^O^+^) (Table 1).

Each separated REE group was clear and had no tailing of the neighboring fractions during measurement by using the MC-ICP-MS. Two-step cation-exchange column chromatographic processes using Biorad AG50W X-8 resin (200–400 mesh) and diluted HCl and HIBA solutions are summarized in Table 2. A 2 M stock solution of HIBA was adjusted to pH 4.6 using ultrapure NH_4_OH (Merck). Nuryono et al. (1998) showed that the elution time and resolution during HIBA column chromatography depended on the pH of HIBA. Therefore, the authors recommended that pH 4.6 is optimal for REE separation by HIBA column chromatography because at pH < 4.6, the eluent is too weak to elute REE ions and at pH > 4.6, the resolution is too low to guarantee adequate REE separation.

**TABLE 2:** Ion chromatography procedure for group separation of REE in this study, modified from Tanaka et al. (2018).

Major composition removal (HCl column)		
Resin: 8 mL AG50W-X8 (cation) 200–400 mesh		
Step	Eluent (UP HCl)	Volume (mL)
Column wash	6 N HCl	20
Column wash	MillQ water	20
Column conditioning	2 N HCl	5
Load sample	2 N HCl	4
Discard major ions	2 N HCl	35
Collect Sr	2 N HCl	35
Collect REE	6 N HCl	40
Colum wash (re-use)	6 N HCl	20
Dry 6 N HCl collected REE fraction		
Second column procedure: group separation of REE to eliminate oxide ions for MC-ICP-MS		
Resin: 0.8 mL AG50W-X8 (neutralized by NH4OH) 200–400 mesh		
Step	Eluent (HIBA)	Volume (mL)
Column wash	0.3 M HIBA	5
Column wash	MillQ water	5
Column conditioning	0.05 M HIBA	5
Sample loading	50 μL DIW	
Column wash	0.045 M HIBA	0.5
Collect HREE	0.09 M HIBA	9
Collect MREE	0.15 M HIBA	15
Collect LREE	0.3 M HIBA	20
Discard resin and wash column for the new sample		

**FIGURE 1:**
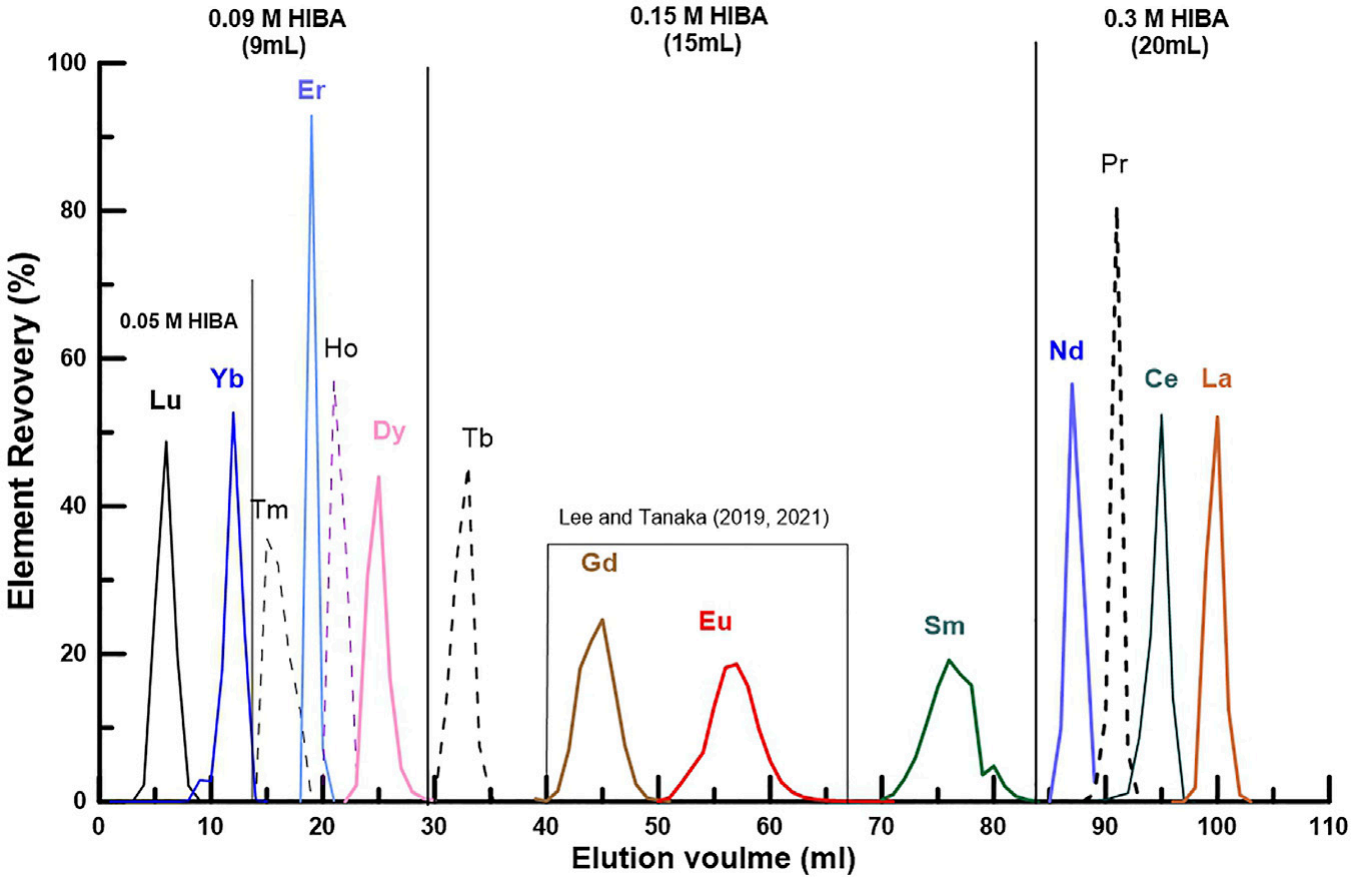
Elution diagram of the REEs using a quartz glass column (ø 3 mm, length 98 mm) filled with 0.8 mL of the cation-exchange resin (Biorad AG 50WX-8, 200–400 mesh) and 2-hydroxyisobutyric acid (HIBA, pH 4.6) as the eluant (Supplementary Table S1). As can be seen in the figure, the elution range for each group (LREE, MREE and HREE) in this study was separately indicated by the concentration and amount of HIBA. In addition, as evident in the MREE part, it is possible to separate each of the 14 REEs with ultra-high purity for isotope ratio determination.

Before dividing the REEs into three groups, the range for separating each of 14 REEs consisting of La to Lu was first determined (Figure 1). Based on the data of Figure 1, we modified the REE fractions as follows: LREE (La, Ce, Pr, and Nd), MREE (Sm, Eu, Gd, and Tb), and HREE (Dy, Ho, Er, Tm, Yb, and Lu) (Table 2). In addition, by controlling the HIBA concentration as shown in Figure 1, it becomes possible to separate each of the 14 REEs with ultra-high purity for isotope ratio determination (Lee and Tanaka, 2019; Lee and Tanaka, 2021).

The HIBA contained in the eluted group REE fraction was dried at 140°C using a hot plate and decomposed with 2% HNO_3_ for MC-ICP-MS analysis.

### 2.3 Instrumentation and MC-ICP-MS analysis of group REEs

Isotope analysis to determine the concentration of each REE was carried out on a ThermoFisher Scientific Neptune Plus MC-ICP-MS equipped with nine Faraday cups and six ion counters at the Korea Institute of Geoscience and Mineral Resources (KIGAM), Daejeon, Korea. Nine Faraday collectors were used for measuring REE concentrations using the standard sample bracketing technique. All of the Faraday collectors are connected to amplifiers with 10^11^ Ω amplifier channels.

In the natural water sample, the measurements were performed in low-resolution mode with Ni Jet-sampling and Ni X-skimmer cones. The samples were introduced into the MC-ICP-MS through a Cetac ARIDUS II™ desolvation system with ultrapure Ar and N_2_ gases used as carrier and sweep gasses to improve the elemental sensitivity. In SRM samples, the measurements were performed in low-resolution mode with the Ni X-sampling and Ni X-skimmer cones in the static mode.

The gain on each Faraday cup was monitored daily to ensure normalization of its efficiency. Operating conditions and data acquisition parameters including cup configuration are shown in Table 3. Twenty measurements were carried out for each sample solution.

**TABLE 3:** Operating conditions for the Neptune MC-ICP-MS.

**Instrument setting**										
RF power (W)	1200									
Plasma Ar gas flow rate (L/min)	16									
Auxiliary Ar gas flow rate (L/min)	1									
Ar carrier gas flow rate (L/min)	1.04									
Sample cone	Jet cone, Ni, 1.2 mm orifice (dry plasma condition, water sample)									
	X-cone, nickel, 0.8 mm orifice (wet plasma condition, rock sample)									
Skimmer cone	X-cone; platinum, 0.8 mm orifice (dry plasma condition, water sample)									
	X-cone; nickel, 0.8 mm orifice (wet plasma condition, rock sample)									
Wash time	100∼120 s									
Lens settings	Optimized for maximum analyte signal intensity, flat-topped peaks, and stability									

**Data acquisition parameters**										
Scan type	Static measurements									
Configuration	Cups	L4	L3	L2	L1	Axial	H1	H2	H3	H4
	LREE	^138^Ba	^139^La	^140^Ce	^141^Pr	^143^Nd	^145^Nd	^146^Nd	^147^Sm	^149^Sm
	MREE	^146^Nd	^147^Sm	^149^Sm	^151^Eu	^153^Eu	^155^Gd	^157^Gd	^159^Tb	^162^Dy
	HREE	^159^Tb	^161^Dy	^163^Dy	^165^Ho	^167^Er	^169^Tm	^172^Yb	^173^Yb	^175^Lu
Zoom optics	Focus quad: 3∼5 V and dispersion quad: 0 V									
Sensitivity	0.88 V/ppb for ^139^La, 1.5 V/ppb for ^151^Eu and 2.1 V/ppb for ^169^Tm (dry plasma condition)									
	0.10 V/ppb for ^139^La, 0.07 V/ppb for ^151^Eu and 0.154 V/ppb for ^169^Tm (wet plasma condition)									
Integration time	4.19 s									
Number of integrations	1									
Cycles/block	20									

There was no polyatomic interference for the selected isotopes of each REE group (LREE, MREE, and HREE) during MC-ICP-MS analysis. The standard solutions used in determining elemental concentrations of the unknown sample were gravimetrically prepared from a single-element solution of the 14 REEs that contained 1 μg/g for atomic analysis (PerkinElmer, Inc). For comparison with the PerkinElmer STD, we also prepared additional standard solutions for LREE, MREE, and HREE from single-element solution of the 14 REEs that contained 100 μg/g of AccuStandard. The concentrations of each REE group (LREE, MREE, and HREE) were certified by a diluted solution of PerkinElmer Multi-Elements Standard (No. N9300232). The linearity of the calibration curve of the beam intensity, according to the concentration change from the home-made PerkinElmer STD and the Accu STD, showed a correlation coefficient value of one on the same line, which indicates that the beam intensity of each REE isotope increases constantly according to the change in the concentration of each REE (Supplementary Figure S1).

The standard sample bracketing method in the MC-ICP-MS is usually used for the determination of isotope ratio variation. We applied it to determine the REE concentration of the geological material.

The concentrations of REEs in SLRS-6 using the MC-ICP-MS were determined based on a standard sample bracketing technique developed by Pourmand et al. (2012) and calculated according to the following equation:

$${Conc}_{sam}={Conc}_{std}\times \left({\mathrm{Int}}_{sam}/{\mathrm{Int}}_{std}\right),$$ (1)

where Conc_sam_ and Conc_std_ indicate the concentration of each REE in the sample and the standard solution, respectively. Int_sam_ and Int_std_ indicate the intensities of the ion beams registered at the Faraday detectors.

For comparison, the REE concentrations in an aliquot prepared from the same stock solution, as measured by using the MC-ICP-MS, were also measured using conventional ICP-MS (NexION350, Perkin Elmer) at KIGAM.

## 3 Results and discussion

### 3.1 Procedural blanks and oxide interferences in the REE measurements by MC-ICP-MS analysis

The primary obstacle in achieving high accuracy and precision in this study is the contribution of total procedure blanks through column chromatography and dilution for MC-ICP-MS analysis. The background equivalent concentration (BEC), as the full instrument background for REEs, was less than 0.2 pg/L (D in Table 4). The total procedural blanks during HCl and HIBA column chromatography in this study are presented with the total acid blank in Table 4.

**TABLE 4:** Total procedural blank and each acid blank during this study.

	Element	Isotopic abundance (%)	Intensity of each isotope in 10 μg/L standard solution (V)	Intensity of each isotope in total procedural blank^a^ (A + B + C + D)(V)	(A) Intensity of each isotope in HCl column resin after wash (V)	(B) Intensity of each isotope in the diluted HCl solution for column chromatography(V)	(C) Intensity of each isotope during HIBA column chromatography(V)	(D) Intensity of each isotope in 2% HNO_3_ for dilution(V)
LREE	^139^La	99.91119	10.77038	0.00034	0.00021	0.00004	0.00002	0.00007
	^140^Ce	88.449	10.71713	0.00041	0.00022	0.00005	0.00005	0.00008
	^141^Pr	100	12.48451	0.00040	0.00024	0.00010	0.00001	0.00005
	^143^Nd	12.173	1.50556	0.00013	0.00004	0.00004	0.00003	0.00002
	^144^Nd	23.973	1.04510	0.00015	0.00004	0.00009	0.00001	0.00001
	^145^Nd	8.293	2.18619	0.00010	0.00006	0.00003	0.00000	0.00001
MREE	^147^Sm	15	1.85969	0.00023	0.00012	0.00008	0.00000	0.00003
	^149^Sm	13.82	1.73922	0.00025	0.00011	0.00010	0.00002	0.00003
	^151^Eu	47.81	6.37159	0.00029	0.00012	0.00014	0.00001	0.00001
	^153^Eu	52.19	7.07577	0.00042	0.00014	0.00021	0.00003	0.00003
	^155^Gd	14.8	1.60256	0.00208	0.00018	0.00183	0.00002	0.00003
	^157^Gd	15.65	1.71856	0.00087	0.00020	0.00062	0.00001	0.00003
	^159^Tb	100	11.91731	0.00027	0.00016	0.00008	0.00000	0.00003
HREE	^161^Dy	18.889	2.49196	0.00079	0.00022	0.00050	0.00000	0.00005
	^163^Dy	24.896	3.34679	0.00094	0.00027	0.00064	0.00001	0.00002
	^165^Ho	100	13.25172	0.00074	0.00021	0.00053	0.00001	-0.00002
	^167^Er	22.869	3.05776	0.00049	0.00012	0.00034	0.00003	0.00001
	^169^Tm	100	16.11966	0.00043	0.00011	0.00031	0.00000	0.00001
	^172^Yb	21.686	4.62514	0.00046	0.00010	0.00036	0.00000	0.00000
	^173^Yb	16.103	3.44865	0.00039	0.00008	0.00027	0.00000	0.00004
	^175^Lu	97.401	14.76457	0.00038	0.00009	0.00028	0.00001	0.00000

^a^ Total procedural blank value in the acids (70 mL 2 N HCl + 40 mL 6 N HCl + 10 mL 0.09 M HIBA + 15 mL 0.15 M HIBA + 15 mL 0.3 M HIBA) used during HCl and HIBA column chromatography.

The blank intensity of each REE isotope in the resin before sample loading was less than 0.0002 V. The blank intensity of each REE isotope in the total amounts of HCl and HIBA solutions used for column chromatography was less than 0.0006 V and 0.00005 V, respectively. Therefore, most of the REE concentration in the acids used during column chromatography was less than 5 pg/L. Blank corrections were performed for each REE group by subtracting the total procedural blank from each REE measurement.

**FIGURE 2:**
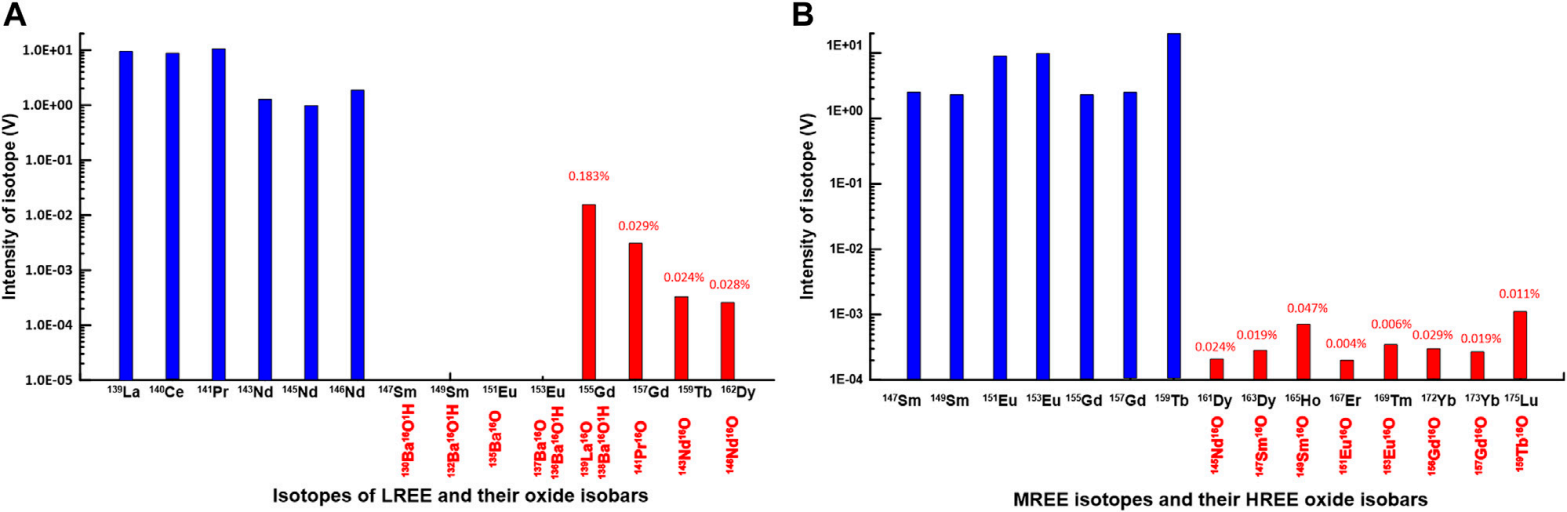
**(A)** Peak intensity (V) of LREE metal isotopes and their oxides in 10 ppb LREE solutions **(B)** Peak intensity (V) of MREE metal isotopes and their oxides in 10 ppb MREE solutions (see Table 1). The “% numbers” indicate % of oxide isobar produced by LREE and MREE standard solutions during MC-ICP-MS (see Table 1).

The Ba concentrations in natural water are generally three orders of magnitude higher than that of Eu, which can generate spectral interferences that cannot be ignored. The oxides that were formed from the LREE and MREE fractions during the measurement using the MC-ICP-MS and the Aridus II desolvating nebulizer system in this study are shown in Figure 2. With the exception of ^139^La^16^O^+^, which is an isobar of ^155^Gd^+^, most oxides from LREE and MREE were formed in less than 0.02% of the primary peak. Therefore, Figure 2 indicates clearly that REE determination by using the MC-ICP-MS and the Aridus II desolvating nebulizer system also may need a correction related to the presence of oxides and hydroxides for measuring REE analysis using the MC-ICP-MS without group separation. However, in this study, we confirmed that the signal of Ba in the MREE fraction was not detected and most of the LREE fraction included a trace of Ba. We, therefore, could neglect Ba oxide interferences and did not need mathematical correction for the results of MREE, including Eu, in the present work.

### 3.2 Reproducibility and accuracy of REE determinations in the water sample

At present, there are no recommended values of REE concentrations for the natural river water reference material SLRS-6 (NRC-CNRS). Recently, Yeghicheyan et al. (2019) reported a valuable compilation of the uncertified REE concentrations in SLRS-6 based on analysis by nine different laboratories. In addition, Babechuk et al. (2020) reported an improved dataset for REE concentrations in SLRS-6. In this article, we used these compiled data for comparison with our determined REE data. The analytical results for REEs in SLRS-6 obtained in the present work are summarized in Table 5 along with a set of compiled data (the values reported) in the references.

**TABLE 5:** Concentration (μg/L) of rare earth elements in SLRS-6 CRM.

Sample weight of ca. 50 mL SLRS-6	50.3	50.5	50.8	50.1	51.0	52.4	50.4	Average	% RSD^a^	Compiled data (Yeghicheyan et al., 2019)	Babechuk et al. (2020)
	(gr)										
La	0.2406	0.2595	0.2635	0.2567	0.2675	0.2695	0.2756	0.2618	4.3217	0.2483	0.2504
Ce	0.2974	0.2943	0.2928	0.3506	0.3597	0.3558	0.3715	0.3317	10.5833	0.2927	0.3002
Pr	0.0608	0.0606	0.0588	0.0658	0.0673	0.0657	0.0689	0.0640	6.0411	0.0591	0.0606
Nd	0.2346	0.2446	0.2402	0.2760	0.2824	0.2710	0.2845	0.2619	8.1436	0.2278	0.2302

Sm	0.0423	0.0386	0.0342	0.0363	0.0425	0.0422	0.0447	0.0401	9.4741	0.0395	0.0385
Eu	0.0065	0.0068	0.0057	0.0060	0.0071	0.0070	0.0074	0.0066	8.9272	0.0073	0.0067
Gd	0.0329	0.0320	0.0319	0.0312	0.0365	0.0362	0.0383	0.0341	8.2007	0.0316	0.0305
Tb	0.0040	0.0040	0.0035	0.0040	0.0046	0.0044	0.0047	0.0042	10.1246	0.0041	0.0039
Dy	0.0184	0.0217	0.0209	0.0204	0.0219	0.0231	0.0250	0.0216	9.6767	0.0219	0.0211
Ho	0.0040	0.0045	0.0045	0.0040	0.0044	0.0045	0.0047	0.0044	5.7940	0.0043	0.0043
Er	0.0127	0.0133	0.0133	0.0129	0.0130	0.0135	0.0136	0.0132	2.4322	0.0124	0.0119
Tm	0.0019	0.0019	0.0019	0.0018	0.0018	0.0018	0.0018	0.0019	3.1604	0.0018	0.0017
Yb	0.0119	0.0115	0.0115	0.0117	0.0106	0.0110	0.0111	0.0113	4.0005	0.0112	0.0112
Lu	0.0020	0.0018	0.0018	0.0020	0.0017	0.0017	0.0019	0.0018	5.3520	0.0019	0.0018
Sr^b^	n.d.^c^	n.d.	n.d.	39.88	39.33	40.63	38.71	39.64	2.05	41.03	-
87Sr/86Sr	-	-	-	0.712092 ± 0.000004(2SE)	0.712090 ± 0.000004(2SE)	0.712107 ± 0.000003(2SE)	0.712114 ± 0.000003(2SE)			0.712051	

^a^ %RSD, relative standard deviation (100 × SD/average) for multiple analyses of the same sample for each element.

^b^ Certified concentration value from the National Research Council Canada (NRC): 40.66 ± 0.32 (μg/L).

^c^ n.d.: not determined.

Table 5 indicates that the observed values for Sm, Dy, Ho, and Yb in the SLRS-6 water sample agreed with the data reported by Yeghicheyan et al. (2019) and Babechuk et al. (2020). Particularly, concentrations of Sm, Eu, Dy, Ho, Yb, and Lu agreed with the data from Babechuk et al. (2020). However, our LREE data appear to be higher than those reported by Babechuk et al. (2020) and Yeghicheyan et al. (2019).

**FIGURE 3:**
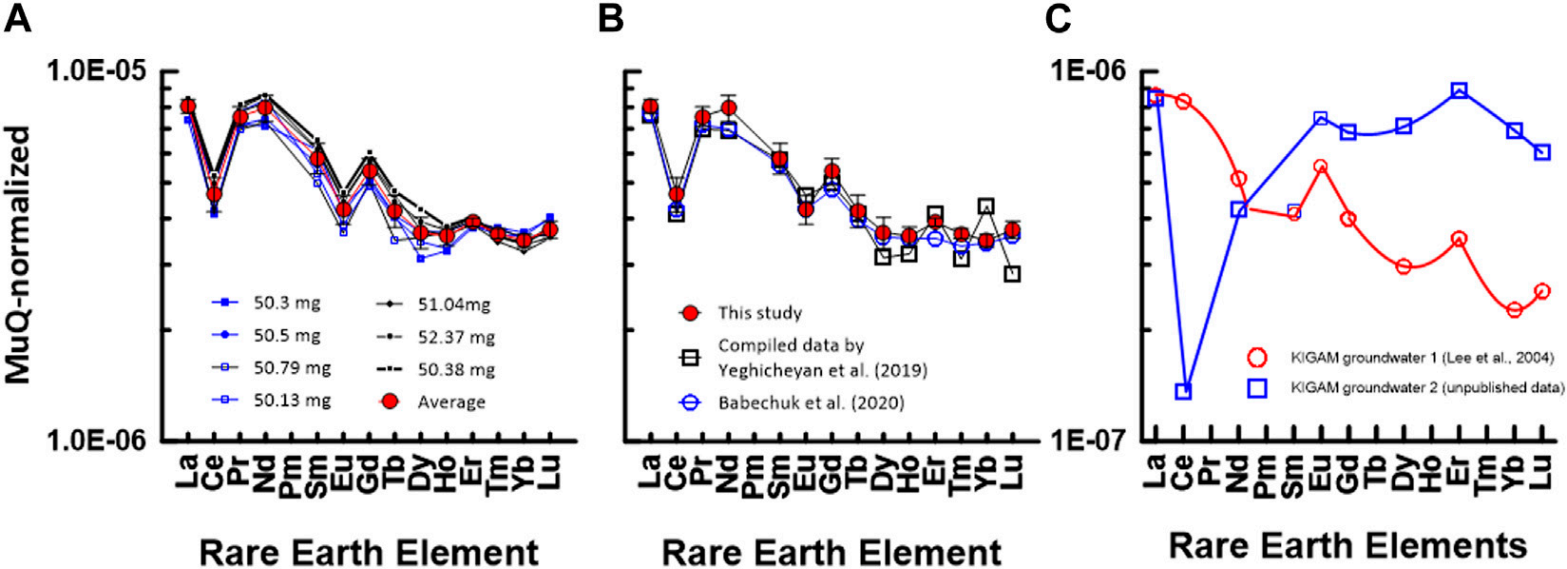
MuQ (Kamber et al., 2005)-normalized REE patterns of SLRS-6: **(A)** in this study and **(B)** from data by Babechuk et al. (2020) and Yeghicheyan et al. (2019). The numbers in **(A)** indicate the gravity weight of ca. 50 mL of SLRS-6 used for preconcentration. **(C)** MuQ-normalized REE patterns of KIGAM groundwaters. REE concentrations in groundwater were measured by ID-TIMS method using a JEOL JMS 05-RB TIMS (Lee et al., 2004).

Comparative REE data (Table 5; Figure 3) enabled us to comment on the accuracy of our method, which depends on accurate preconcentration and perfect recovery of the REE fraction during column chromatography. The concentrations of Eu, Dy, and Lu in this study were slightly lower than the compiled values presented by Yeghicheyan et al. (2019), whereas those of the other REEs show a higher value. However, when our data were compared with those of Babechuk et al. (2020), the concentrations of Sm, Eu, Dy, Ho, Yb, and Lu were similar and the others were slightly higher. The recovery values for REEs obtained in this study were all quite close to 100%, with standard deviations under 2% (Table 5). These results support the conclusion that the present results for REEs in SLRS-6 are also accurate.

The concentrations of REEs in SLRS-6 in this study cover a range from approximately 300 pg/mL of La, Ce, and Nd to approximately 2 pg/mL of Tm and Lu. The concentration data of each REE group obtained by our technique were reproduced to ∼ 5% accuracy. The reproducibility of the REE concentration can be further improved by enhanced column chromatography and cup configuration.

### 3.3 Reproducibility and accuracy of REE determinations in rock samples

At present, there are a lot of REE concentrations of the SRMs as geochemical reference rocks (Kawabe, 1995; Liang et al., 2000; Jochum et al., 2015). In this article, we used these compiled data for comparison with our REE data in the C1 chondrite-normalized REE pattern. The analytical results for REEs of the SRMs obtained in this work are summarized in Table 6 along with the recommended values and the reported values in the literature. Table 6 also includes the data determined by the conventional ICP-MS method for comparison.

**TABLE 6:** Concentration (μg/L) of rare earth elements in JB1b, JG2, and JA1 Standard Rock Materials.

		Decomposed sample weight (mg)	Used sample weight (mg)	La	Ce	Pr	Nd	Sm	Eu	Gd	Tb	Dy	Ho	Er	Tm	Yb	Lu	Srb	87Sr/86Sr
JB1b	Conventional ICP-MS: no column chromatography	29.9	10.0	39.02	67.79	7.17	26.14	4.97	1.505	4.80	0.68	4.05	0.79	2.20	0.31	2.03	0.30	431.96	-
		49.7	9.98	39.70	68.26	7.28	26.61	5.03	1.503	4.89	0.68	3.98	0.78	2.18	0.30	2.01	0.29	435.29	-
		99.9	9.96	38.99	67.20	7.16	26.28	4.97	1.483	4.86	0.68	4.00	0.78	2.21	0.31	2.02	0.30	430.32	-
	Average	-	-	39.24	67.75	7.20	26.34	4.99	1.497	4.85	0.68	4.01	0.79	2.20	0.31	2.02	0.30	432.52	-
	% RSD^a^	-	-	1.02	0.78	0.93	0.92	0.64	0.814	0.98	0.34	0.88	0.54	0.64	1.70	0.58	1.17	0.59	-
	Conventional ICP-MS: after HCl column chromatography	29.9	10.0	33.75	59.93	6.71	24.72	4.73	1.37	4.30	0.64	3.78	0.71	2.08	0.29	1.90	0.28	434.13	-
		49.7	10.0	34.91	61.68	6.81	25.04	4.80	1.38	4.42	0.66	3.73	0.72	2.08	0.29	1.92	0.28	361.05	-
		99.9	10.0	35.32	61.91	6.66	25.29	4.76	1.36	4.45	0.66	3.75	0.71	2.04	0.29	1.89	0.28	413.04	-
	Average	-	-	34.66	61.17	6.73	25.02	4.76	1.371	4.39	0.65	3.76	0.71	2.07	0.29	1.90	0.28	402.74	-
	% RSD^a^	-	-	2.35	1.77	1.11	1.15	0.74	0.686	1.82	1.44	0.70	0.98	1.13	0.17	0.84	1.27	9.34	-
	MC-ICP-MS	99.9	28.5	38.38	63.08	7.08	26.69	5.11	1.307	4.65	0.70	4.08	0.81	2.24	0.31	2.02	0.28	434.8	0.704097 ± 0.000002(2SE)
	Reference value of GSJ	-	-	37.60	65.90	7.30	26.00	5.07	1.460	4.67	0.69	3.99	0.71	2.18	0.33	2.10	0.33	439.0	0.704098
JG2	Conventional ICP-MS: no column chromatography	50.3	10.0	17.76	44.27	5.78	24.03	7.67	0.082	9.21	1.72	11.80	2.52	7.84	1.17	7.86	1.15	14.15	-
		50.3	10.0	18.30	44.87	5.81	24.00	7.50	0.087	8.84	1.63	11.07	2.37	7.24	1.09	7.33	1.06	14.02	-
		50.0	9.97	18.19	45.15	5.80	23.84	7.32	0.083	8.53	1.62	11.07	2.41	7.41	1.12	7.54	1.08	13.88	-
	Average	-	-	18.08	44.77	5.80	23.96	7.50	0.084	8.86	1.66	11.32	2.43	7.50	1.13	7.57	1.09	14.02	-
	% RSD^a^	-	-	1.60	1.01	0.26	0.44	2.31	3.554	3.85	3.58	3.74	3.03	4.14	3.75	3.52	4.06	0.94	-
	Conventional ICP-MS: after HCl column chromatography	50.3	10.1	19.34	47.64	6.19	26.40	8.54	0.09	9.94	1.84	12.50	2.60	8.22	1.25	8.47	1.22	13.40	-
		50.3	10.0	19.60	48.49	6.33	26.34	8.38	0.09	9.59	1.76	11.82	2.43	7.83	1.17	8.04	1.16	13.07	-
		50.0	10.0	19.60	48.01	6.24	25.62	7.92	0.09	9.32	1.75	11.79	2.48	7.89	1.20	8.00	1.16	13.07	-
	Average	-	-	19.51	48.05	6.25	26.12	8.28	0.087	9.62	1.78	12.04	2.50	7.98	1.21	8.17	1.18	13.18	-
	% RSD^a^	-	-	0.78	0.89	1.18	1.67	3.87	1.215	3.24	2.71	3.36	3.49	2.63	3.38	3.19	3.05	1.43	-
	MC-ICP-MS	10.1	49.0	17.45	40.04	5.36	22.30	7.76	0.095	9.17	1.74	11.44	2.44	6.91	0.88	5.40	0.72	15.7	0.758496 ± 0.000016(2SE)
	Reference value of GSJ	-	-	19.90	48.30	6.20	26.40	7.78	0.100	8.01	1.62	10.50	1.67	6.04	1.16	6.85	1.22	17.9	0.758560
	Liang et al. (2000)	-	-	19.80	50.10	6.29	26.20	8.59	0.100	9.94	1.87	12.90	2.76	8.11	1.28	8.81	1.21	18.5	-
	Kawabe (1995)	-	-	18.60	44.90	5.61	23.90	7.62	0.092	8.88	1.71	11.60	2.48	7.75	1.12	8.00	1.15	-	-
JA1	Conventional ICP-MS: no column chromatography	30.9	10.3	5.13	13.49	2.17	11.10	3.39	1.131	4.30	0.73	4.90	1.05	3.15	0.46	3.07	0.46	256.0	-
		49.8	9.96	4.96	13.07	2.11	10.74	3.33	1.119	4.24	0.71	4.78	1.02	3.05	0.44	3.01	0.44	251.2	-
		50.0	10.0	4.95	13.04	2.07	10.84	3.37	1.142	4.31	0.71	4.82	1.03	3.09	0.45	2.96	0.45	247.8	-
		50.0	4.97	4.96	13.06	2.11	10.89	3.41	1.119	4.26	0.71	4.70	1.01	3.00	0.44	2.93	0.44	248.0	-
		100.0	10.0	4.91	12.97	2.07	10.73	3.37	1.117	4.23	0.70	4.74	1.01	2.99	0.43	2.94	0.44	248.7	-
	Average	-	-	4.98	13.02	2.09	10.81	3.39	1.118	4.24	0.71	4.72	1.01	3.00	0.44	2.94	0.44	248.37	-
	% RSD^a^	-	-	1.67	1.59	1.99	1.41	0.94	0.960	0.88	1.64	1.60	1.83	2.15	1.94	1.94	1.79	1.38	-
	Conventional ICP-MS: after HCl column chromatography	30.9	10.3	4.81	12.84	2.04	10.77	3.36	1.05	3.96	0.70	4.67	0.97	2.96	0.43	2.90	0.44	242.94	-
		49.8	9.90	4.67	12.35	1.94	10.47	3.28	1.04	3.90	0.68	4.52	0.93	2.86	0.42	2.81	0.42	235.26	-
		50.0	10.1	4.75	12.57	2.04	10.58	3.30	1.03	3.90	0.69	4.56	0.96	2.94	0.43	2.87	0.43	233.15	-
		50.0	9.97	4.70	12.45	1.98	10.31	3.32	1.04	3.89	0.69	4.53	0.94	2.91	0.42	2.81	0.42	233.43	-
		100.0	20.2	4.66	12.38	1.98	10.26	3.24	1.03	3.78	0.68	4.49	0.92	2.81	0.41	2.74	0.42	226.99	-
	Average	-	-	4.72	12.42	1.98	10.28	3.28	1.038	3.83	0.68	4.51	0.93	2.86	0.41	2.78	0.42	230.21	-
	% RSD^a^	-	-	1.36	1.58	2.31	2.04	1.39	0.723	1.79	1.32	1.59	1.96	2.17	1.72	2.15	2.13	2.49	-
	MC-ICP-MS	50.0	14.80	5.17	13.13	2.15	11.44	3.67	1.302	4.58	0.79	5.25	1.12	3.16	0.43	2.66	0.36	270.7	0.703574 ± 0.000003(2SE)
	Jochum et al. (2015)	-	-	4.88	13.15	2.08	10.69	3.40	1.112	4.15	0.73	4.75	1.03	2.96	0.45	2.95	0.45	259.3	-
	Reference value of GSJ	-	-	5.24	13.30	1.71	10.90	3.52	1.200	4.36	0.75	4.55	0.95	3.04	0.47	3.03	0.47	263.0	0.703533

^a^ %RSD, relative standard deviation (100 × SD/average) for multiple analyses of the same sample for each element.

#### 3.3.1 JB1b: Geochemical reference material for basalt

**FIGURE 4:**
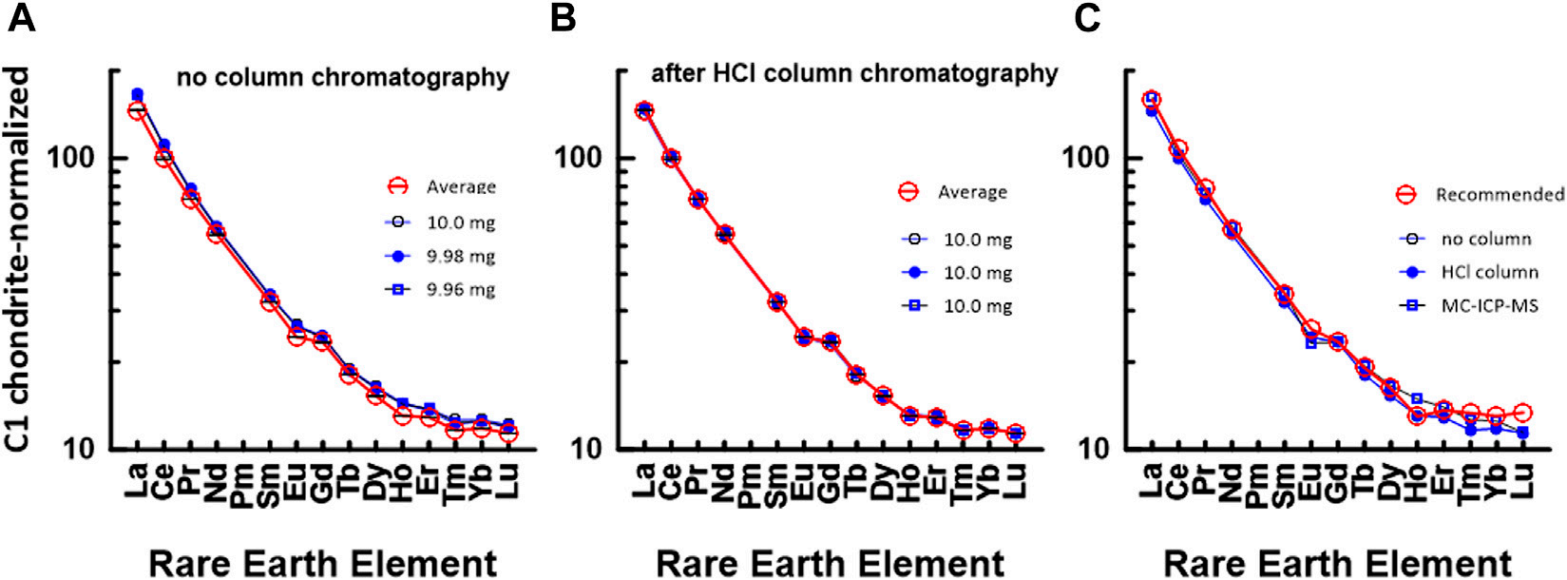
C1 chondrite (McDonough and Sun, 1995)-normalized REE patterns of JB1b: **(A)** measured by conventional ICP-MS method without HCl column chromatography in this study and **(B)** measured by conventional ICP-MS method after HCl column chromatography in this study. The numbers in **(A)** indicate the gravity weight of sample. **(C)** based on the data measured by MC-ICP-MS in this study, recommended value by GSJ, and average value of (a) and (b).

Unlike the environmental samples, the REE concentrations of the rock samples are interpreted relative to those in C1 chondrite (McDonough and Sun, 1995), and this plot method is useful for judging the reliability of the measured REE data. Figure 4 shows a chondrite-normalized REE pattern diagram for JB1b. Figure 4A shows a C1 chondrite-normalized REE plot based on data measured by the conventional ICP-MS technique without HCl column chromatography, whereas Figure 4B shows a plot based on data measured by ICP-MS after HCl column chromatography. Also, Figure 4C shows a comparison diagram based on the average values in Figures 4A, B, the data measured by the MC-ICP-MS, and the recommended values. Except for the HREEs, Figure 4C shows that the measured data in this study and the recommended values agree well. However, the HREE plot from the recommended values in Figure 4C deviated from the smooth curved pattern. This suggests that we may need re-examination of the recommended values from Er to Lu. Nevertheless, the recovery values for most REEs in the JGb1 used in this study, measured using the MC-ICP-MS, were all quite close to 100%, with standard deviations under 2% (Table 6).

#### 3.3.2 JA1: Geochemical reference material for andesite

**FIGURE 5:**
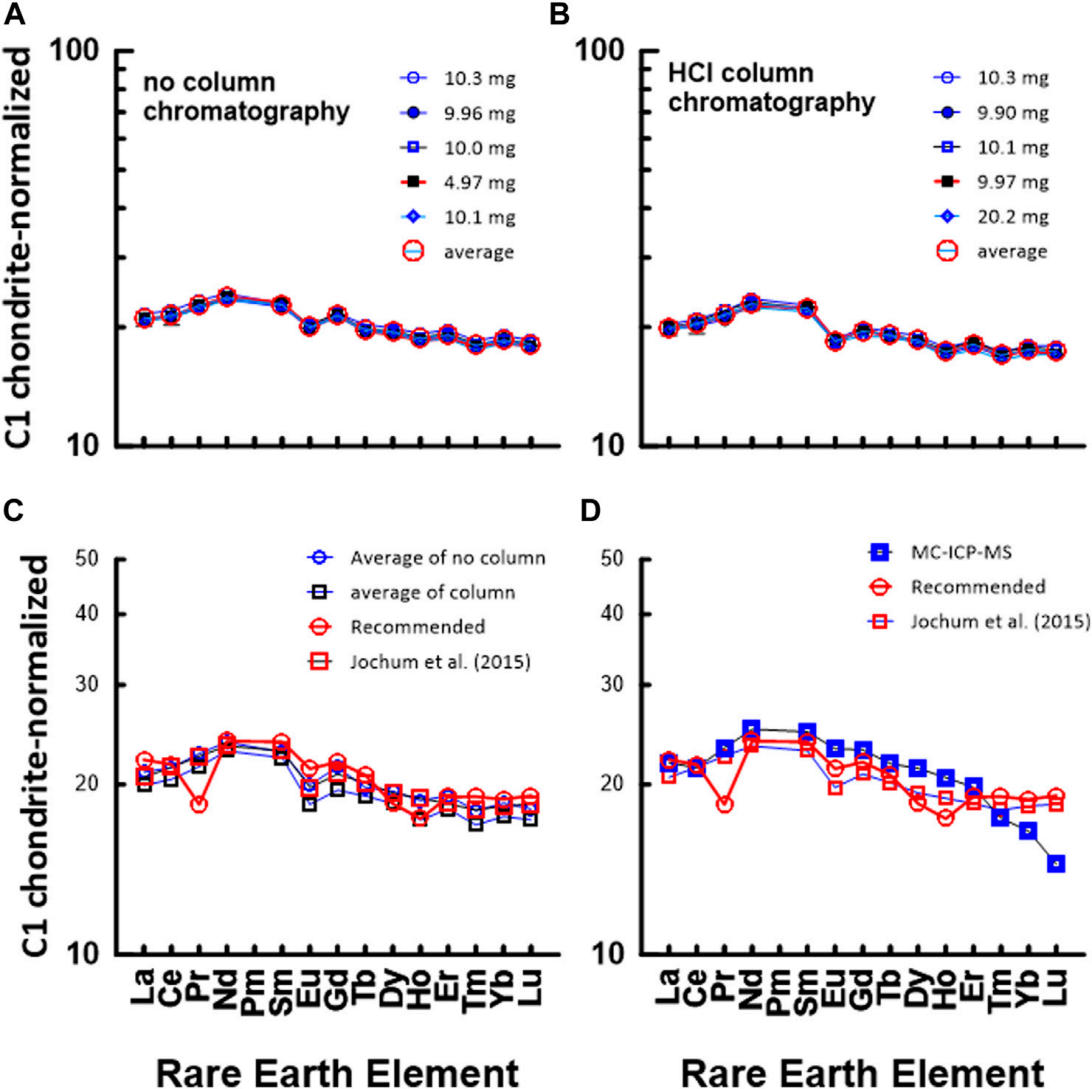
C1 chondrite-normalized REE patterns of JA1: **(A)** measured by conventional ICP-MS method without HCl column chromatography in this study and **(B)** measured by conventional ICP-MS method after HCl column chromatography in this study. The numbers in **(A)** indicate the gravity weight of sample. **(C)** comparison with the data measured by ICP-MS in this study and recommended value by GSJ, **(D)** comparison with the data measured by ICP-MS in this study and recommended value by GSJ.

Figure 5 shows a chondrite-normalized REE pattern diagram for JA1. Similar to Figures 4A, B, Figure 5A shows a C1 chondrite-normalized REE plot based on data measured by the conventional ICP-MS technique without HCl column chromatography, whereas Figure 5B shows a diagram based on data measured by ICP-MS after HCl column chromatography. Also, Figures 5C, D show a comparison between the average values in Figures 5A, B and the data measured by the MC-ICP-MS, respectively, with the recommended values in the literature. Except for Pr and Ho, the chondrite-normalized REE pattern measured by the conventional ICP-MS method in Figure 5C corresponds well with that of the recommended values. However, in Figure 5D, the HREE data obtained using the MC-ICP-MS deviated from other data. During the experiment, no loss of HREE, that is, the HREE content by Sr elutes, was found in the primary separation process by HCl column chromatography. At present, although the cause of the decrease in HREE has not been clearly elucidated, the smooth curve of the HREEs appears to be analytically and geochemically remarkable.

#### 3.3.3 JG2: Geochemical reference material for granite

**FIGURE 6:**
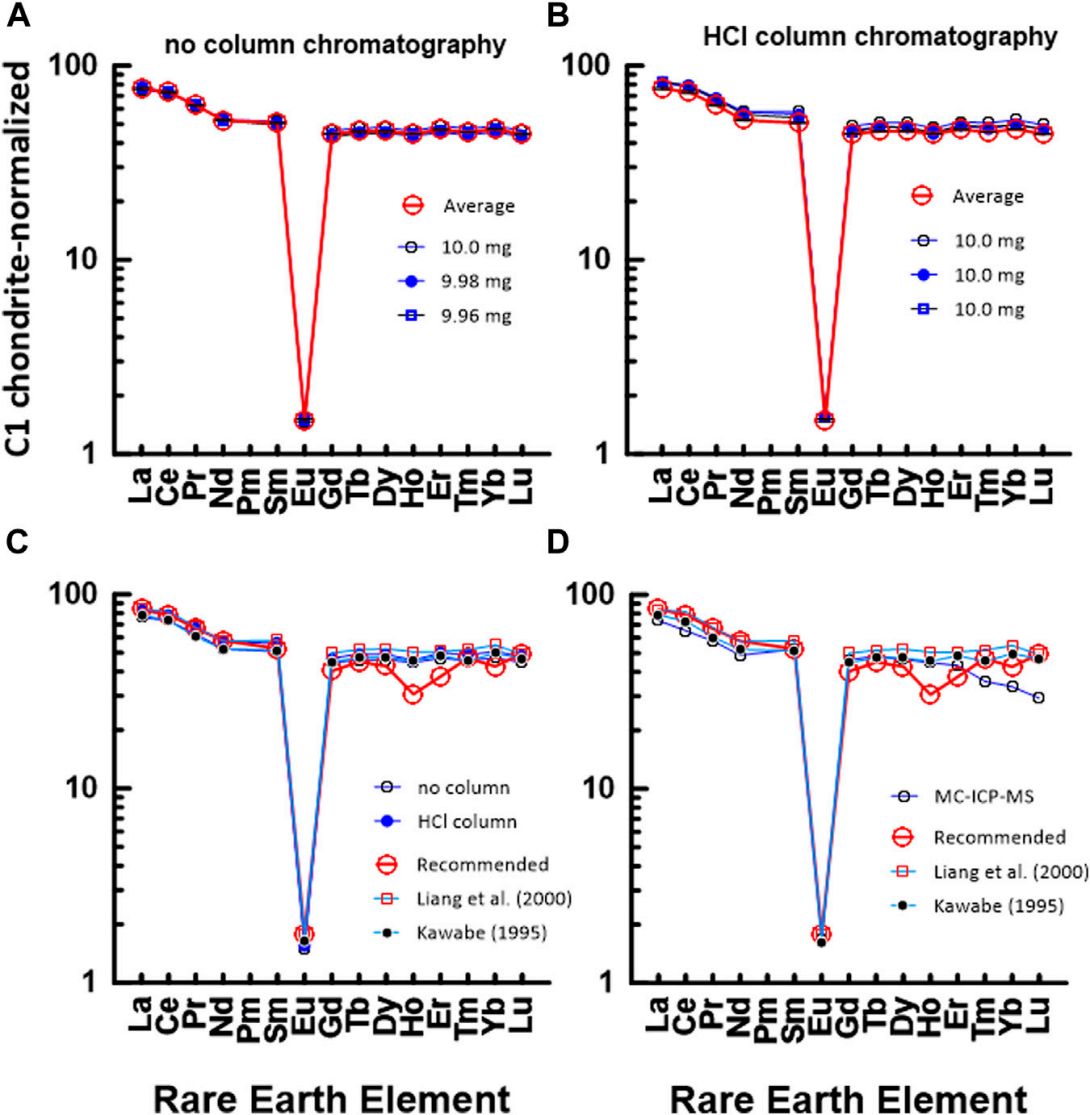
C1 chondrite-normalized REE patterns of JG2: **(A)** measured by conventional ICP-MS method without HCl column chromatography in this study and **(B)** measured by conventional ICP-MS method after HCl column chromatography in this study. The numbers in **(A)** indicate the gravity weight of sample. **(C)** comparison with the data measured by ICP-MS in this study and recommended value by GSJ, **(D)** comparison with the data measured by ICP-MS in this study and recommended value by GSJ.

Figure 6 shows a chondrite-normalized REE pattern diagram for JG2. Similar to Figures 4, 5, Figure 6A shows a CI chondrite-normalized REE plot based on data measured by the conventional ICP-MS technique without HCl column chromatography, whereas Figure 6B shows a diagram based on data measured by ICP-MS after HCl column chromatography. Also, Figures 6C, D show a comparison between the average values of 5a and 5b, the values in the literature, and the measured value by using the MC-ICP-MS in this study. As shown in Figures 6A, C, D, a difference is observed between the HREE pattern based on the recommended value and the HREE pattern based on the data in this study and the literature. This indicates that it is difficult to accurately measure the concentration of rare earth elements in highly differentiated igneous rocks such as JG2. Particularly, when comparing the measured values using the MC-ICP-MS in this study with the recommended values or previously reported values, HREEs had lower values, as in JA1.

### 3.4 Comparison with other techniques for REE determinations

#### 3.4.1 Natural water sample

The REE concentrations in geological and environmental materials are produced by water–rock and water–sediment matrices. The normalized REE pattern, which is directly related to the fractionation or differentiation history of the source material, has an important role in interpretation of the behavior of REEs in geochemical and environmental systems. The rare earth elements display unique properties in which the ionic radius decreases regularly as the atomic number increases and these elements also have similar physical/chemical properties. Therefore, the REE concentrations of geological materials can be evaluated with a pattern normalized to those of a primitive chondrite or an upper continental crust composite such as PAAS (Post-Archean Australian Shale). Such patterns for PAAS materials provide a baseline to evaluate bulk REE fractionation in terms of pattern enrichment or depletion relative to the crust and to determine anomalous element behavior. Recently, Babechuk et al. (2020) used the alluvial sediment composite “Mud from Queensland” (MuQ) (Kamber et al., 2005) for REE normalization in river water. The concentration of REEs in environmental materials including natural water is produced by the water–sediment reaction rather than the water–rock interaction in the crust. This means that MuQ from Kamber et al. (2005) is more appropriate than PAAS as a normalizing criterion for interpreting normalized REE plots in natural waters. Lawrence and Kamber (2006) and Babechuk et al. (2020) applied the “smoothness” of the non-anomalous (or potentially non-anomalous) elements in the normalized pattern (i.e., excluding La, Ce, Eu, Gd, Y, and Lu) to attest to the quality of the data and the accuracy of inter-element REE ratios.

Therefore, in this study, we also used the value of MuQ as a normalizing criterion for interpreting normalized REE plots in SLRS-6. MuQ-normalized REE patterns of SLRS-6 data from this study are shown in Figure 3A. The REE pattern (solid red symbols) was plotted based on the average values of REEs of SLRS-6 obtained during this study. Figure 3B compares the MuQ-normalized REE patterns of SLRS-6 data from the study of Babechuk et al. (2020) and Yeghicheyan et al. (2019). Except for Nd, our data overlap with the data of Babechuk et al. (2020). However, Nd deviated slightly from LREE and showed a relatively higher value. Further experiments are needed to clarify the cause of Nd enrichment.

Figure 3C depicts MuQ-normalized REE patterns of KIGAM groundwaters pumped from a borehole. The REE contents were measured by ID-TIMS (isotope dilution thermal ion mass spectrometry) using a JEOL JMS-05RB thermal ion mass spectrometer (Lee et al., 2004). ID-TIMS is the best method to accurately and precisely measure the concentration of REEs in geological materials, but it cannot measure the concentration of mono-isotope elements such as Pr, Tb, Ho, and Tm. Therefore, we could not compose a complete REE pattern, as shown in Figures 3A, B. Nevertheless, we can deduce a smooth curve of the W-type REE tetrad effect. Moreover, the HREE pattern in Figure 3C shows the REE tetrad effect of W-type (Masuda et al., 1987). Particularly, the smooth HREE pattern of SLRS-6 in this study suggests that it clearly exhibits the ‘smoothness’ typical of non-anomalous elements in the normalized pattern (i.e., W-type of the REE tetrad effect), thereby confirming that the HREE concentration in natural water has a geochemical REE pattern, indicating the W-type REE tetrad effect. Therefore, our data indicate that the MC-ICP-MS measurement method using group separation based on column chromatography will be a good method for the accurate and precise measurement of the 14 REE concentrations, including the four mono-isotope elements Pr, Tb, Ho, and Tm.

#### 3.4.2 Rock samples

As mentioned in Section 3.3.1, the normalized REE pattern of the rock has an important role in the interpretation of the behavior of REEs in the crust–mantle for clarifying the fractionation or differentiation history of the source material during magma evolution. Therefore, we selected and compared the SRMs according to the petrography related to the degree of magmatic differentiation, such as JB1a, JA2, and JG2. JG2 is highly fractionated granite that exhibits the REE tetrad effect. The existence of the REE tetrad effect observed in the chondrite-normalized REE plot is a special phenomenon whose formation mechanism has not been clearly identified (LeeMasuda and Kim, 1994; 2010; 2013; Kawabe, 1995; Monecke et al., 2011; Sami et al., 2022). Therefore, REE data for JG2 were reported by several researchers (Kawabe, 1995; Liang et al., 2000).

##### 3.4.2.1 Sr concentration and isotopic ratio

One of the advantages of column chromatography for REE concentration is the ability to measure the Sr concentration during HCl column chromatography before HIBA column chromatography. Therefore, for the Sr concentration and isotope ratio measurements in SLRS-6, JB1a, JA2, and JG2, we collected the Sr fraction before REE preconcentration and determined the Sr concentration and their isotope ratios under wet plasma conditions using the Neptune MC-ICP-MS (Tables 5, 6). Measurements of the Sr concentration and isotope ratio were performed in low-resolution mode, with Ni normal sampling and Ni X-skimmer cones. Each sample was subjected to 108 cycles (12 cycles/block) with a 4.19 c integration interval. The typical sample aspiration rate was 80∼100 μL/min.

In this study, the measured ^87^Sr/^86^Sr ratios of SLRS-6 range from 0.712090 to 0.712114, with a mean value of 0.712101 (Table 5), indicating that the values were slightly higher than 0.712051, the value determined by Yeghicheyan et al. (2019). Sr concentrations vary from 38.71 to 40.64 μg/L (certified value, 40.66 μg/L), which reflects homogeneous results in our experimental procedures and good recovery.

The concentration and ^87^Sr/^86^Sr ratios of the SRMs such as JGb1b, JA1, and JG2 are shown in Table 6. In this study, the measured Sr concentrations of JGb1, JA1, and JG2 are 434.8 mg/L, 270.7 mg/L, and 15.7 mg/L, respectively. The certified values of Sr concentration of JGb1b, JA1, and JG2 are 439.0 mg/L, 263.0 mg/L, and 17.9 mg/L, respectively. Also, the measured ^87^Sr/^86^Sr ratios of the SRMs such as JGb1b, JA1, and JG2 are 0.704097 ± 0.000002 (2SE), 0.703574 ± 0.000003 (2SE), and 0.758496 ± 0.000016 (2SE), respectively. Moreover, the reference ^87^Sr/^86^Sr ratios of the SRMs such as JGb1b, JA1, and JG2 at the homepage of the Geological Survey of Japan (GSJ) are 0.704098, 0.703533, and 0.758560, respectively. These Sr isotope values in this study are similar to those of reference values reported in the literature. In addition, Sr concentrations of the SRMs determined by using the MC-ICP-MS also were consistent with the recommended or reference values, which reflect homogeneous results in our experimental procedures and good recovery.

## 4 Conclusion

In this study, a method to determine accurately and precisely the REE concentrations in water and rock samples by using the MC-ICP-MS, without oxide interference, has been developed. Before MC-ICP-MS analysis, REEs were divided into three groups: LREEs (La–Ce–Pr–Nd), MREEs (Sm–Eu–Gd–Tb), and HREEs (Dy–Ho–Er–Tm–Yb–Lu). The recovery rate and accuracy of REE concentrations from SLRS-6 CRM and JB1b, JA1, and JG2 SRMs in this study indicate good agreement with the REE concentration in SLRS-6, JB1b, JG2, and JA1 from the literature. The recovery rate and accuracy of REE concentrations from SRMs for rock materials in this study also indicate good agreement with the REE concentration by ICP-MS data from the literature, except for HREEs. Our results indicate that the accuracy and precision of our method can help to interpret the behavior of REEs in natural water systems as well as rock materials. In particular, it will become one of the main concentration measurement methods for REEs that can lead to a clearer understanding of the unique geochemical behavior of REEs, namely, the REE tetrad effect. In addition, accurate and precise determination of Sr concentration and isotope ratio in natural water and SRMs for rock samples was obtained as a by-product during these investigations.

## Data Availability

The original contributions presented in the study are included in the article/Supplementary Material; further inquiries can be directed to the corresponding author.
