# Supplementary material for: Development of an analytical method for accurate and precise determination of rare earth element concentrations in geological materials using an MC-ICP-MS and group separation
Source: Front Chem. 2023 Jan 12;10:906160. doi: 10.3389/fchem.2022.906160 (PMC9878377; doi:10.3389/fchem.2022.906160)
Supplement: Supplementary file 1 [file DataSheet1.PDF]

Supplementary Table S1. Recovery rate of each REE measured by ICP-QMS (NexION 350, Perkin Elmer Lt

| Eluted sample No. | Sample Id | amounts of eluted HIBA (mL) | La 138 (%) | Ce 140 (%) | Pr 141 (%) | Nd 146 (%) | Sm 147 (%) |
|---|---|---|---|---|---|---|---|
| 1 | 0.05M | 1 | 0.0 | 0.0 | 0.0 | 1.0 | 3.0 |
| 2 | 0.05M | 1 | 0.0 | 0.0 | 0.0 | 0.0 | 5.0 |
| 3 | 0.05M | 1 | 0.0 | 0.0 | 0.0 | 2.0 | 1.0 |
| 4 | 0.05M | 1 | 0.0 | 0.0 | 0.0 | 1.0 | 2.0 |
| 5 | 0.05M | 1 | 0.0 | 0.0 | 0.0 | 7.0 | 0.0 |
| 6 | 0.05M | 1 | 0.0 | 0.0 | 0.0 | 0.0 | 2.0 |
| 7 | 0.05M | 1 | 0.0 | 0.0 | 0.0 | 1.0 | 2.0 |
| 8 | 0.05M | 1 | 0.0 | 0.0 | 0.0 | 0.0 | 1.0 |
| 9 | 0.05M | 1 | 0.0 | 0.0 | 0.0 | 4.0 | 1.0 |
| 10 | 0.05M | 1 | 0.0 | 0.0 | 0.0 | 3.0 | 1.0 |
| 11 | 0.05M | 1 | 0.0 | 0.0 | 0.0 | 2.0 | 0.0 |
| 12 | 0.05M | 1 | 0.0 | 0.0 | 0.0 | 1.0 | 3.0 |
| 13 | 0.05M | 1 | 0.0 | 0.0 | 0.0 | 2.0 | 2.0 |
| 14 | 0.05M | 1 | 0.0 | 0.0 | 0.0 | 3.0 | 3.0 |
| 15 | 0.09M | 1 | 0.0 | 0.0 | 0.0 | 2.0 | 2.0 |
| 16 | 0.09M | 1 | 0.0 | 0.0 | 0.0 | 2.0 | 2.0 |
| 17 | 0.09M | 1 | 0.0 | 0.0 | 0.0 | 2.0 | 4.0 |
| 18 | 0.09M | 1 | 0.0 | 0.0 | 0.0 | 2.0 | 4.0 |
| 19 | 0.09M | 1 | 0.0 | 0.0 | 0.0 | 9.3 | 2.3 |
| 20 | 0.09M | 1 | 0.0 | 0.0 | 0.0 | 4.0 | 2.3 |
| 21 | 0.09M | 1 | 0.0 | 0.0 | 0.0 | 8.7 | 4.0 |
| 22 | 0.09M | 1 | 0.0 | 0.0 | 0.0 | 4.3 | 3.7 |
| 23 | 0.09M | 1 | 0.0 | 0.0 | 0.0 | 5.0 | 2.3 |
| 24 | 0.09M | 1 | 0.0 | 0.0 | 0.0 | 5.0 | 6.3 |
| 25 | 0.09M | 1 | 0.0 | 0.0 | 0.0 | 10.7 | 5.0 |
| 26 | 0.09M | 1 | 0.0 | 0.0 | 0.0 | 7.0 | 4.3 |
| 27 | 0.09M | 1 | 0.0 | 0.0 | 0.0 | 7.0 | 6.0 |
| 28 | 0.09M | 1 | 0.0 | 0.0 | 0.0 | 5.3 | 3.0 |
| 29 | 0.09M | 1 | 0.0 | 0.0 | 0.0 | 4.3 | 2.3 |
| 30 | 0.09M | 1 | 0.0 | 0.0 | 0.0 | 5.7 | 4.3 |
| 31 | 0.09M | 1 | 0.0 | 0.0 | 0.0 | 6.0 | 2.0 |
| 32 | 0.09M | 1 | 0.0 | 0.0 | 0.0 | 2.0 | 0.0 |
| 33 | 0.09M | 1 | 0.0 | 0.0 | 0.0 | 2.0 | 0.0 |
| 34 | 0.09M | 1 | 0.0 | 0.0 | 0.0 | 2.0 | 2.0 |
| 35 | 0.09M | 1 | 0.0 | 0.0 | 0.0 | 1.0 | 0.0 |
| 36 | 0.09M | 1 | 0.0 | 0.0 | 0.0 | 0.0 | 2.0 |
| 37 | 0.09M | 1 | 0.0 | 0.0 | 0.0 | 2.0 | 2.0 |
| 38 | 0.09M | 1 | 0.0 | 0.0 | 0.0 | 0.0 | 3.0 |
| 39 | 0.09M | 1 | 0.0 | 0.0 | 0.0 | 5.0 | 6.0 |
| 40 | 0.09M | 1 | 0.0 | 0.0 | 0.0 | 1.0 | 1.0 |
| 41 | 0.12M | 1 | 0.0 | 0.0 | 0.0 | 12.0 | 2.0 |
| 42 | 0.12M | 1 | 0.0 | 0.0 | 0.0 | 17.0 | 2.0 |
| 43 | 0.12M | 1 | 0.0 | 0.0 | 0.0 | 10.0 | 3.0 |

| | | | | | | | |
|---|---|---|---|---|---|---|---|
| 44 | 0.12M | 1 | 0.0 | 0.0 | 0.0 | 45.0 | 1.0 |
| 45 | 0.12M | 1 | 0.0 | 0.0 | 0.0 | 47.0 | 3.0 |
| 46 | 0.12M | 1 | 0.0 | 0.0 | 0.0 | 40.0 | 4.0 |
| 47 | 0.12M | 1 | 0.0 | 0.0 | 0.0 | 46.0 | 3.0 |
| 48 | 0.12M | 1 | 0.0 | 0.0 | 0.0 | 36.0 | 5.0 |
| 49 | 0.12M | 1 | 0.0 | 0.0 | 0.0 | 38.0 | 3.0 |
| 50 | 0.12M | 1 | 0.0 | 0.0 | 0.0 | 35.0 | 6.0 |
| 51 | 0.12M | 1 | 0.1 | 0.0 | 0.0 | 120.0 | 18.0 |
| 52 | 0.12M | 1 | 0.0 | 0.0 | 0.0 | 40.0 | 5.0 |
| 53 | 0.12M | 1 | 0.0 | 0.0 | 0.0 | 66.0 | 9.0 |
| 54 | 0.12M | 1 | 0.0 | 0.0 | 0.0 | 25.0 | 3.0 |
| 55 | 0.12M | 1 | 0.0 | 0.0 | 0.0 | 32.0 | 0.0 |
| 56 | 0.12M | 1 | 0.0 | 0.0 | 0.0 | 20.0 | 0.0 |
| 57 | 0.12M | 1 | 0.1 | 0.0 | 0.0 | 104.0 | 0.0 |
| 58 | 0.12M | 1 | 0.0 | 0.0 | 0.0 | 64.0 | 0.0 |
| 59 | 0.12M | 1 | 0.0 | 0.0 | 0.0 | 14.0 | 0.0 |
| 60 | 0.12M | 1 | 0.0 | 0.0 | 0.0 | 20.0 | 0.0 |
| 61 | 0.12M | 1 | 0.0 | 0.0 | 0.0 | 20.0 | 0.0 |
| 62 | 0.12M | 1 | 0.0 | 0.0 | 0.0 | 55.0 | 0.0 |
| 63 | 0.12M | 1 | 0.0 | 0.0 | 0.0 | 42.0 | 0.0 |
| 64 | 0.12M | 1 | 0.1 | 0.0 | 0.0 | 0.0 | 0.0 |
| 65 | 0.12M | 1 | 0.0 | 0.0 | 0.0 | 0.0 | 0.0 |
| 66 | 0.12M | 1 | 0.0 | 0.0 | 0.0 | 0.0 | 0.0 |
| 67 | 0.12M | 1 | 0.1 | 0.0 | 0.0 | 0.0 | 0.0 |
| 68 | 0.12M | 1 | 0.0 | 0.0 | 0.0 | 0.0 | 0.0 |
| 69 | 0.12M | 1 | 0.0 | 0.0 | 0.0 | 0.0 | **0.1** |
| 70 | 0.12M | 1 | 0.0 | 0.0 | 0.0 | 0.0 | **0.3** |
| 71 | 0.12M | 1 | 0.0 | 0.0 | 0.0 | 0.0 | **1.0** |
| 72 | 0.12M | 1 | 0.0 | 0.0 | 0.0 | 0.0 | **3.0** |
| 73 | 0.12M | 1 | 0.0 | 0.0 | 0.0 | 0.0 | **6.0** |
| 74 | 0.12M | 1 | 0.0 | 0.0 | 0.0 | 0.0 | **10.6** |
| 75 | 0.12M | 1 | 0.0 | 0.0 | 0.0 | 0.0 | **15.5** |
| 76 | 0.12M | 1 | 0.0 | 0.0 | 0.0 | 0.0 | **19.1** |
| 77 | 0.12M | 1 | 0.0 | 0.0 | 0.0 | 0.0 | **17.2** |
| 78 | 0.12M | 1 | 0.0 | 0.0 | 0.0 | 0.0 | **15.8** |
| 79 | 0.12M | 1 | 0.0 | 0.0 | 0.0 | 0.0 | **3.6** |
| 80 | 0.12M | 1 | 0.0 | 0.0 | 0.0 | 0.0 | **4.8** |
| 81 | 0.12M | 1 | 0.0 | 0.0 | 0.0 | 0.0 | **2.0** |
| 82 | 0.12M | 1 | 0.0 | 0.0 | 0.0 | 0.0 | **0.7** |
| 83 | 0.12M | 1 | 0.0 | 0.0 | 0.0 | 0.0 | **0.2** |
| 84 | 0.12M | 1 | 0.0 | 0.0 | 0.0 | 0.0 | **0.1** |
| 85 | 0.2M | 1 | 0.0 | 0.0 | 0.0 | **0.0** | 0.0 |
| 86 | 0.2M | 1 | 0.0 | 0.0 | 0.0 | **9.9** | 0.0 |
| 87 | 0.2M | 1 | 0.0 | 0.0 | 0.0 | **56.5** | 0.0 |
| 88 | 0.2M | 1 | 0.0 | 0.0 | **0.1** | **30.9** | 0.0 |
| 89 | 0.2M | 1 | 0.0 | **0.2** | **1.0** | **2.7** | 0.0 |
| 90 | 0.3M | 1 | 0.0 | **0.2** | **11.5** | 0.0 | 0.0 |
| 91 | 0.3M | 1 | 0.0 | **0.7** | **80.7** | 0.0 | 0.0 |

| | | | | | | | |
|---|---|---|---|---|---|---|---|
| 92 | 0.3M | 1 | 0.0 | **1.1** | **6.7** | 0.0 | 0.0 |
| 93 | 0.3M | 1 | 0.0 | **8.7** | 0.0 | 0.0 | 0.0 |
| 94 | 0.3M | 1 | 0.0 | **22.1** | 0.0 | 0.0 | 0.0 |
| 95 | 0.3M | 1 | 0.0 | **52.4** | 0.0 | 0.0 | 0.0 |
| 96 | 0.3M | 1 | 0.0 | **14.2** | 0.0 | 0.0 | 0.0 |
| 97 | 0.3M | 1 | 0.0 | **0.4** | 0.0 | 0.0 | 0.0 |
| 98 | 0.3M | 1 | **1.9** | 0.0 | 0.0 | 0.0 | 0.0 |
| 99 | 0.3M | 1 | **32.8** | 0.0 | 0.0 | 0.0 | 0.0 |
| 100 | 0.3M | 1 | **52.1** | 0.0 | 0.0 | 0.0 | 0.0 |
| 101 | 0.3M | 1 | **12.4** | 0.0 | 0.0 | 0.0 | 0.0 |
| 102 | 0.3M | 1 | **0.9** | 0.0 | 0.0 | 0.0 | 0.0 |
| 103 | 0.3M | 1 | 0.0 | 0.0 | 0.0 | 0.0 | 0.0 |
| | SUM | | 100.0 | 100.1 | 100.0 | 100.0 | 100.0 |

l.) for each eluted fracton during HIBA Column calibration

| Recovery rate (%) | | | | | | | |
|---|---|---|---|---|---|---|---|
| Eu 153 (%) | Gd 157 (%) | Tb 159 (%) | Dy 163 (%) | Ho 165 (%) | Er 166 (%) | Tm 169 (%) | Yb 174 (%) |
| 0.0 | 0.0 | 0.0 | 0.0 | 0.0 | 0.0 | 0.0 | 0.0 |
| 0.0 | 0.0 | 0.0 | 0.0 | 0.0 | 0.0 | 0.0 | 0.0 |
| 0.0 | 0.0 | 0.0 | 0.0 | 0.0 | 0.0 | 0.0 | 0.0 |
| 0.0 | 0.0 | 0.0 | 0.0 | 0.0 | 0.0 | 0.0 | 0.0 |
| 0.0 | 0.0 | 0.0 | 0.0 | 0.0 | 0.0 | 0.0 | 0.0 |
| 0.0 | 0.0 | 0.0 | 0.0 | 0.0 | 0.0 | 0.0 | 0.0 |
| 0.0 | 0.0 | 0.0 | 0.0 | 0.0 | 0.0 | 0.0 | 0.0 |
| 0.0 | 0.0 | 0.0 | 0.0 | 0.0 | 0.0 | 0.0 | **0.2** |
| 0.0 | 0.0 | 0.0 | 0.0 | 0.0 | 0.0 | 0.0 | **2.9** |
| 0.0 | 0.0 | 0.0 | 0.0 | 0.0 | 0.0 | 0.0 | **2.8** |
| 0.0 | 0.0 | 0.0 | 0.0 | 0.0 | 0.0 | 0.0 | **17.7** |
| 0.0 | 0.0 | 0.0 | 0.0 | 0.0 | 0.0 | 0.0 | **52.7** |
| 0.0 | 0.0 | 0.0 | 0.0 | 0.0 | 0.0 | 0.0 | **23.5** |
| 0.0 | 0.0 | 0.0 | 0.0 | 0.0 | 0.0 | 0.0 | **0.2** |
| 0.0 | 0.0 | 0.0 | 0.0 | 0.0 | 0.0 | **35.7** | **0.0** |
| 0.0 | 0.0 | 0.0 | 0.0 | 0.0 | 0.0 | **32.1** | 0.0 |
| 0.0 | 0.0 | 0.0 | 0.0 | 0.0 | 0.0 | **19.9** | 0.0 |
| 0.0 | 0.0 | 0.0 | 0.0 | 0.0 | 0.0 | **12.2** | 0.0 |
| 0.0 | 0.0 | 0.0 | 0.0 | 0.0 | **92.9** | **0.1** | 0.0 |
| 0.0 | 0.0 | 0.0 | 0.0 | **2.2** | **7.0** | 0.0 | 0.0 |
| 0.0 | 0.0 | 0.0 | 0.0 | **56.9** | **0.1** | 0.0 | 0.0 |
| 0.0 | 0.0 | 0.0 | 0.0 | **36.3** | 0.0 | 0.0 | 0.0 |
| 0.0 | 0.0 | 0.0 | **2.2** | **4.6** | 0.0 | 0.0 | 0.0 |
| 0.0 | 0.0 | 0.0 | **30.6** | 0.0 | 0.0 | 0.0 | 0.0 |
| 0.0 | 0.0 | 0.0 | **44.0** | 0.0 | 0.0 | 0.0 | 0.0 |
| 0.0 | 0.0 | 0.0 | **16.8** | 0.0 | 0.0 | 0.0 | 0.0 |
| 0.0 | 0.0 | 0.0 | **4.5** | 0.0 | 0.0 | 0.0 | 0.0 |
| 0.0 | 0.0 | 0.0 | **1.3** | 0.0 | 0.0 | 0.0 | 0.0 |
| 0.0 | 0.0 | 0.0 | **0.4** | 0.0 | 0.0 | 0.0 | 0.0 |
| 0.0 | 0.0 | 0.0 | **0.2** | 0.0 | 0.0 | 0.0 | 0.0 |
| 0.0 | 0.0 | **0.1** | 0.0 | 0.0 | 0.0 | 0.0 | 0.0 |
| 0.0 | 0.0 | **3.5** | 0.0 | 0.0 | 0.0 | 0.0 | 0.0 |
| 0.0 | 0.0 | **25.0** | 0.0 | 0.0 | 0.0 | 0.0 | 0.0 |
| 0.0 | 0.0 | **43.9** | 0.0 | 0.0 | 0.0 | 0.0 | 0.0 |
| 0.0 | 0.0 | **22.8** | 0.0 | 0.0 | 0.0 | 0.0 | 0.0 |
| 0.0 | 0.0 | **4.4** | 0.0 | 0.0 | 0.0 | 0.0 | 0.0 |
| 0.0 | 0.0 | **0.3** | 0.0 | 0.0 | 0.0 | 0.0 | 0.0 |
| 0.0 | 0.0 | 0.0 | 0.0 | 0.0 | 0.0 | 0.0 | 0.0 |
| 0.0 | 0.0 | 0.0 | 0.0 | 0.0 | 0.0 | 0.0 | 0.0 |
| 0.0 | 0.0 | 0.0 | 0.0 | 0.0 | 0.0 | 0.0 | 0.0 |
| 0.0 | 0.0 | 0.0 | 0.0 | 0.0 | 0.0 | 0.0 | 0.0 |
| 0.0 | 0.0 | 0.0 | 0.0 | 0.0 | 0.0 | 0.0 | 0.0 |
| 0.0 | **2.2** | 0.0 | 0.0 | 0.0 | 0.0 | 0.0 | 0.0 |

| | | | | | | | |
|---|---|---|---|---|---|---|---|
| 0.0 | **13.8** | 0.0 | 0.0 | 0.0 | 0.0 | 0.0 | 0.0 |
| 0.0 | **27.3** | 0.0 | 0.0 | 0.0 | 0.0 | 0.0 | 0.0 |
| 0.0 | **26.4** | 0.0 | 0.0 | 0.0 | 0.0 | 0.0 | 0.0 |
| 0.0 | **15.5** | 0.0 | 0.0 | 0.0 | 0.0 | 0.0 | 0.0 |
| 0.0 | **8.3** | 0.0 | 0.0 | 0.0 | 0.0 | 0.0 | 0.0 |
| 0.0 | **4.5** | 0.0 | 0.0 | 0.0 | 0.0 | 0.0 | 0.0 |
| 0.0 | **2.0** | 0.0 | 0.0 | 0.0 | 0.0 | 0.0 | 0.0 |
| 0.0 | 0.0 | 0.0 | 0.0 | 0.0 | 0.0 | 0.0 | 0.0 |
| **0.2** | 0.0 | 0.0 | 0.0 | 0.0 | 0.0 | 0.0 | 0.0 |
| **1.6** | 0.0 | 0.0 | 0.0 | 0.0 | 0.0 | 0.0 | 0.0 |
| **7.8** | 0.0 | 0.0 | 0.0 | 0.0 | 0.0 | 0.0 | 0.0 |
| **15.2** | 0.0 | 0.0 | 0.0 | 0.0 | 0.0 | 0.0 | 0.0 |
| **18.7** | 0.0 | 0.0 | 0.0 | 0.0 | 0.0 | 0.0 | 0.0 |
| **10.3** | 0.0 | 0.0 | 0.0 | 0.0 | 0.0 | 0.0 | 0.0 |
| **12.0** | 0.0 | 0.0 | 0.0 | 0.0 | 0.0 | 0.0 | 0.0 |
| **11.0** | 0.0 | 0.0 | 0.0 | 0.0 | 0.0 | 0.0 | 0.0 |
| **8.2** | 0.0 | 0.0 | 0.0 | 0.0 | 0.0 | 0.0 | 0.0 |
| **4.2** | 0.0 | 0.0 | 0.0 | 0.0 | 0.0 | 0.0 | 0.0 |
| **4.0** | 0.0 | 0.0 | 0.0 | 0.0 | 0.0 | 0.0 | 0.0 |
| **2.8** | 0.0 | 0.0 | 0.0 | 0.0 | 0.0 | 0.0 | 0.0 |
| **1.7** | 0.0 | 0.0 | 0.0 | 0.0 | 0.0 | 0.0 | 0.0 |
| **1.3** | 0.0 | 0.0 | 0.0 | 0.0 | 0.0 | 0.0 | 0.0 |
| **0.9** | 0.0 | 0.0 | 0.0 | 0.0 | 0.0 | 0.0 | 0.0 |
| 0.0 | 0.0 | 0.0 | 0.0 | 0.0 | 0.0 | 0.0 | 0.0 |
| 0.0 | 0.0 | 0.0 | 0.0 | 0.0 | 0.0 | 0.0 | 0.0 |
| 0.0 | 0.0 | 0.0 | 0.0 | 0.0 | 0.2 | 0.0 | 0.0 |
| 0.0 | 0.0 | 0.0 | 0.0 | 0.0 | 0.0 | 0.0 | 0.0 |
| 0.0 | 0.0 | 0.0 | 0.0 | 0.0 | 0.0 | 0.0 | 0.0 |
| 0.0 | 0.0 | 0.0 | 0.0 | 0.0 | 0.0 | 0.0 | 0.0 |
| 0.0 | 0.0 | 0.0 | 0.0 | 0.1 | 0.0 | 0.0 | 0.0 |
| 0.0 | 0.0 | 0.0 | 0.0 | 0.1 | 0.0 | 0.0 | 0.0 |
| 0.0 | 0.0 | 0.0 | 0.0 | 0.2 | 0.0 | 0.0 | 0.0 |
| 0.0 | 0.0 | 0.0 | 0.0 | 0.2 | 0.0 | 0.0 | 0.0 |
| 0.0 | 0.0 | 0.0 | 0.0 | 0.2 | 0.0 | 0.0 | 0.0 |
| 0.0 | 0.0 | 0.0 | 0.0 | 0.2 | 0.0 | 0.0 | 0.0 |
| 0.0 | 0.0 | 0.0 | 0.0 | 0.0 | 0.0 | 0.0 | 0.0 |
| 0.0 | 0.0 | 0.0 | 0.0 | 0.0 | 0.0 | 0.0 | 0.0 |
| 0.0 | 0.0 | 0.0 | 0.0 | 0.0 | 0.0 | 0.0 | 0.0 |
| 0.0 | 0.0 | 0.0 | 0.0 | 0.0 | 0.0 | 0.0 | 0.0 |
| 0.0 | 0.0 | 0.0 | 0.0 | 0.0 | 0.0 | 0.0 | 0.0 |
| 0.0 | 0.0 | 0.0 | 0.0 | 0.0 | 0.0 | 0.0 | 0.0 |
| 0.0 | 0.0 | 0.0 | 0.0 | 0.0 | 0.0 | 0.0 | 0.0 |
| 0.0 | 0.0 | 0.0 | 0.0 | 0.0 | 0.0 | 0.0 | 0.0 |
| 0.0 | 0.0 | 0.0 | 0.0 | 0.0 | 0.0 | 0.0 | 0.0 |
| 0.0 | 0.0 | 0.0 | 0.0 | 0.0 | 0.0 | 0.0 | 0.0 |
| 0.0 | 0.0 | 0.0 | 0.0 | 0.0 | 0.0 | 0.0 | 0.0 |
| 0.0 | 0.0 | 0.0 | 0.0 | 0.0 | 0.0 | 0.0 | 0.0 |
| 0.0 | 0.0 | 0.0 | 0.0 | 0.0 | 0.0 | 0.0 | 0.0 |

| | | | | | | | |
|---|---|---|---|---|---|---|---|
| 0.0 | 0.0 | 0.0 | 0.0 | 0.0 | 0.0 | 0.0 | 0.0 |
| 0.0 | 0.0 | 0.0 | 0.0 | 0.0 | 0.0 | 0.0 | 0.0 |
| 0.0 | 0.0 | 0.0 | 0.0 | 0.0 | 0.0 | 0.0 | 0.0 |
| 0.0 | 0.0 | 0.0 | 0.0 | 0.0 | 0.0 | 0.0 | 0.0 |
| 0.0 | 0.0 | 0.0 | 0.0 | 0.0 | 0.0 | 0.0 | 0.0 |
| 0.0 | 0.0 | 0.0 | 0.0 | 0.0 | 0.0 | 0.0 | 0.0 |
| 0.0 | 0.0 | 0.0 | 0.0 | 0.0 | 0.0 | 0.0 | 0.0 |
| 0.0 | 0.0 | 0.0 | 0.0 | 0.0 | 0.0 | 0.0 | 0.0 |
| 0.0 | 0.0 | 0.0 | 0.0 | 0.0 | 0.0 | 0.0 | 0.0 |
| 0.0 | 0.0 | 0.0 | 0.0 | 0.0 | 0.0 | 0.0 | 0.0 |
| 0.0 | 0.0 | 0.0 | 0.0 | 0.0 | 0.0 | 0.0 | 0.0 |
| 0.0 | 0.0 | 0.0 | 0.0 | 0.0 | 0.0 | 0.0 | 0.0 |
| 100.0 | 100.0 | 100.0 | 100.0 | 100.0 | 100.0 | 100.0 | 100.0 |

| | | | | | Ion beam ir |
|---|---|---|---|---|---|
| Lu 175 (%) | La 138 (cps) | Ce 140 (cps) | Pr 141 (cps) | Nd 146 (cps) | Sm 147 (cps) |
| 0.0 | 339.0 | 4.0 | 4.0 | 1.0 | 3.0 |
| 0.0 | 403.0 | 3.0 | 1.0 | 0.0 | 5.0 |
| 0.0 | 309.0 | 4.0 | 1.0 | 2.0 | 1.0 |
| **2.2** | 295.0 | 2.0 | 4.0 | 1.0 | 2.0 |
| **26.6** | 304.0 | 5.0 | 4.0 | 7.0 | 0.0 |
| **48.8** | 285.0 | 4.0 | 3.0 | 0.0 | 2.0 |
| **19.8** | 303.0 | 0.0 | 1.0 | 1.0 | 2.0 |
| **2.1** | 243.0 | 3.0 | 5.0 | 0.0 | 1.0 |
| **0.2** | 248.0 | 2.0 | 2.0 | 4.0 | 1.0 |
| **0.2** | 277.0 | 3.0 | 4.0 | 3.0 | 1.0 |
| **0.1** | 242.0 | 2.0 | 2.0 | 2.0 | 0.0 |
| **0.1** | 262.0 | 4.0 | 6.0 | 1.0 | 3.0 |
| 0.0 | 261.0 | 5.0 | 1.0 | 2.0 | 2.0 |
| 0.0 | 297.0 | 4.0 | 1.0 | 3.0 | 3.0 |
| 0.0 | 282.0 | 0.0 | 6.0 | 2.0 | 2.0 |
| 0.0 | 330.0 | 4.0 | 3.0 | 2.0 | 2.0 |
| 0.0 | 263.0 | 8.0 | 4.0 | 2.0 | 4.0 |
| 0.0 | 250.0 | 1.0 | 4.0 | 2.0 | 4.0 |
| 0.0 | 44 | 83 | 15 | 9 | 2 |
| 0.0 | 37 | 46 | 13 | 4 | 2 |
| 0.0 | 65 | 83 | 18 | 9 | 4 |
| 0.0 | 32 | 39 | 9 | 4 | 4 |
| 0.0 | 22 | 41 | 10 | 5 | 2 |
| 0.0 | 27 | 46 | 9 | 5 | 6 |
| 0.0 | 63 | 92 | 19 | 11 | 5 |
| 0.0 | 48 | 100 | 11 | 7 | 4 |
| 0.0 | 24 | 51 | 10 | 7 | 6 |
| 0.0 | 31 | 37 | 9 | 5 | 3 |
| 0.0 | 21 | 30 | 6 | 4 | 2 |
| 0.0 | 18 | 35 | 6 | 6 | 4 |
| 0.0 | 1052.0 | 10.0 | 4.0 | 6.0 | 2.0 |
| 0.0 | 900.0 | 4.0 | 3.0 | 2.0 | 0.0 |
| 0.0 | 1008.0 | 4.0 | 4.0 | 2.0 | 0.0 |
| 0.0 | 874.0 | 5.0 | 1.0 | 2.0 | 2.0 |
| 0.0 | 967.0 | 4.0 | 0.0 | 1.0 | 0.0 |
| 0.0 | 940.0 | 6.0 | 2.0 | 0.0 | 2.0 |
| 0.0 | 807.0 | 8.0 | 1.0 | 2.0 | 2.0 |
| 0.0 | 1169.0 | 2.0 | 3.0 | 0.0 | 3.0 |
| 0.0 | 835.0 | 4.0 | 1.0 | 5.0 | 6.0 |
| 0.0 | 1426.1 | 2.0 | 1.0 | 1.0 | 1.0 |
| 0.0 | 230.0 | 56.0 | 3.0 | 12.0 | 2.0 |
| 0.0 | 273.0 | 48.0 | 6.0 | 17.0 | 2.0 |
| 0.0 | 198.0 | 62.0 | 7.0 | 10.0 | 3.0 |

| | | | | | |
|---|---|---|---|---|---|
| 0.0 | 331.0 | 60.0 | 10.0 | 45.0 | 1.0 |
| 0.0 | 193.0 | 50.0 | 9.0 | 47.0 | 3.0 |
| 0.0 | 191.0 | 74.0 | 8.0 | 40.0 | 4.0 |
| 0.0 | 200.0 | 54.0 | 2.0 | 46.0 | 3.0 |
| 0.0 | 267.0 | 85.0 | 11.0 | 36.0 | 5.0 |
| 0.0 | 340.0 | 95.0 | 13.0 | 38.0 | 3.0 |
| 0.0 | 287.0 | 97.0 | 11.0 | 35.0 | 6.0 |
| 0.0 | 3417.7 | 845.0 | 162.0 | 120.0 | 18.0 |
| 0.0 | 885.0 | 232.0 | 45.0 | 40.0 | 5.0 |
| 0.0 | 1549.1 | 352.0 | 61.0 | 66.0 | 9.0 |
| 0.0 | 405.0 | 117.0 | 18.0 | 25.0 | 3.0 |
| 0.0 | 914.0 | 262.0 | 44.0 | 32.0 | 6.0 |
| 0.0 | 475.0 | 155.0 | 24.0 | 20.0 | 5.0 |
| 0.0 | 3361.5 | 867.0 | 153.0 | 104.0 | 17.0 |
| 0.0 | 1578.2 | 415.0 | 63.0 | 64.0 | 13.0 |
| 0.0 | 283.0 | 92.0 | 11.0 | 14.0 | 6.0 |
| 0.0 | 197.0 | 84.0 | 6.0 | 20.0 | 5.0 |
| 0.0 | 589.0 | 109.0 | 12.0 | 20.0 | 1.0 |
| 0.0 | 1584.2 | 418.0 | 74.0 | 55.0 | 6.0 |
| 0.0 | 1341.1 | 362.0 | 63.0 | 42.0 | 6.0 |
| 0.0 | 2355.4 | 584.0 | 98.0 | 76.0 | 16.0 |
| 0.0 | 174.0 | 58.0 | 8.0 | 13.0 | 3.0 |
| 0.0 | 188.0 | 83.0 | 9.0 | 22.0 | 5.0 |
| 0.0 | 3208.4 | 180.0 | 8.0 | 18.0 | 5.0 |
| 0.0 | 62.0 | 68.0 | 14.0 | 8.0 | 180.0 |
| 0.0 | 132.0 | 66.0 | 36.0 | 38.0 | **1090.0** |
| 0.0 | 66.0 | 64.0 | 10.0 | 16.0 | **5377.0** |
| 0.0 | 64.0 | 62.0 | 14.0 | 16.0 | **18990.6** |
| 0.0 | 46.0 | 84.0 | 10.0 | 26.0 | **60250.8** |
| 0.0 | 82.0 | 56.0 | 18.0 | 18.0 | **118019.5** |
| 0.0 | 38.0 | 72.0 | 20.0 | 12.0 | **210292.5** |
| 0.0 | 46.0 | 66.0 | 14.0 | 20.0 | **307412.4** |
| 0.0 | 70.0 | 84.0 | 10.0 | 16.0 | **379235.8** |
| 0.0 | 48.0 | 66.0 | 10.0 | 8.0 | **341389.0** |
| 0.0 | 70.0 | 92.0 | 10.0 | 26.0 | **312028.9** |
| 0.0 | 34.0 | 82.0 | 16.0 | 10.0 | **71546.7** |
| 0.0 | 50.0 | 74.0 | 12.0 | 22.0 | **94904.2** |
| 0.0 | 62.0 | 100.0 | 22.0 | 18.0 | **40316.8** |
| 0.0 | 68.0 | 70.0 | 12.0 | 2.0 | **13694.6** |
| 0.0 | 42.0 | 64.0 | 8.0 | 6.0 | **4646.8** |
| 0.0 | 68.0 | 110.0 | 20.0 | 14.0 | **1152.0** |
| 0.0 | 66 | 622 | 77 | **451** | **1,980,347** |
| 0.0 | 54 | 857 | 142 | **308752** | 32 |
| 0.0 | 59 | 1180 | 278 | **1764541** | 52 |
| 0.0 | 66 | 1651 | **3427** | **964269** | 46 |
| 0.0 | 56 | **4885** | **57457** | **83395** | 26 |
| 0.0 | 52.0 | **7427.9** | **637932.4** | **3,121,407.1** | 14.0 |
| 0.0 | 80.0 | **21496.2** | **4473293.6** | 60.0 | 8.0 |

| | | | | | |
|---|---|---|---|---|---|
| 0.0 | 32.0 | **34519.7** | **371607.2** | 20.0 | 2.0 |
| 0.0 | 60.0 | **260823.5** | **5,543,717.3** | 12.0 | 0.0 |
| 0.0 | 84.0 | **664911.9** | 520.0 | 18.0 | 4.0 |
| 0.0 | 40.0 | **1574021.8** | 218.0 | 6.0 | 6.0 |
| 0.0 | 30.0 | **425520.4** | 106.0 | 4.0 | 2.0 |
| 0.0 | 66.0 | **11264.4** | 112.0 | 14.0 | 4.0 |
| 0.0 | **66397** | **3,004,871** | 46 | 17 | 4 |
| 0.0 | **1163015** | 499 | 22 | 6 | 3 |
| 0.0 | **1844734** | 311 | 25 | 9 | 0 |
| 0.0 | **437716** | 280 | 27 | 9 | -4 |
| 0.0 | **31455** | 218 | 28 | 11 | 3 |
| 0.0 | **3,543,317** | 674 | 26 | 7 | 2 |
| 100.0 | | | | | |

ıtensities of REE isoyopes measured by ICP-QMS(NexION 350)

| Eu 153 (cps) | Gd 157 (cps) | Tb 159 (cps) | Dy 163 (cps) | Ho 165 (cps) | Er 166 (cps) | Tm 169 (cps) | Yb 174 (cps) |
|---|---|---|---|---|---|---|---|
| 6.0 | 13.0 | 3.0 | 1.0 | 1.0 | 0.0 | 1.0 | 5.0 |
| 1.0 | 16.0 | 0.0 | 1.0 | 2.0 | 0.0 | 1.0 | 0.0 |
| 5.0 | 4.0 | 1.0 | 0.0 | 2.0 | 3.0 | 2.0 | 3.0 |
| 3.0 | 3.0 | 1.0 | 1.0 | 2.0 | 1.0 | 2.0 | 1.0 |
| 8.0 | 10.0 | 0.0 | 1.0 | 0.0 | 0.0 | 2.0 | 0.0 |
| 2.0 | 6.0 | 2.0 | 4.0 | 0.0 | 1.0 | 3.0 | 1.0 |
| 7.0 | 2.0 | 1.0 | 1.0 | 0.0 | 1.0 | 0.0 | 40.0 |
| 3.0 | 9.0 | 0.0 | 1.0 | 2.0 | 0.0 | 2.0 | **1105.0** |
| 8.0 | 5.0 | 0.0 | 1.0 | 2.0 | 1.0 | 2.0 | **16791.9** |
| 3.0 | 5.0 | 1.0 | 1.0 | 1.0 | 4.0 | 4.0 | **16096.1** |
| 5.0 | 10.0 | 2.0 | 2.0 | 0.0 | 0.0 | 0.0 | **102958.7** |
| 5.0 | 6.0 | 2.0 | 0.0 | 1.0 | 2.0 | 2.0 | **306302.9** |
| 5.0 | 5.0 | 2.0 | 2.0 | 1.0 | 1.0 | 2.0 | **136765.5** |
| 4.0 | 9.0 | 1.0 | 2.0 | 2.0 | 2.0 | 55.0 | **1268.1** |
| 7.0 | 3.0 | 0.0 | 3.0 | 1.0 | 1.0 | **323966.6** | **115.0** |
| 5.0 | 5.0 | 2.0 | 0.0 | 1.0 | 0.0 | **291781.7** | **581,403.2** |
| 2.0 | 5.0 | 0.0 | 1.0 | 0.0 | 3.0 | **180720.0** | 3.0 |
| 7.0 | 8.0 | 0.0 | 1.0 | 1.0 | 1.0 | **111001.6** | 4.0 |
| 4 | 3 | 3 | 5 | 34 | **354,353** | **1,048** | 91 |
| 3 | 4 | 2 | 7 | **11,003** | **26,722** | **908,518** | 36 |
| 5 | 5 | 8 | 5 | **279,537** | **456** | 26 | 18 |
| 3 | 5 | 2 | 44 | **177,963** | **381,530** | 11 | 12 |
| 4 | 3 | 2 | **12,532** | **22,383** | 35 | 9 | 9 |
| 3 | 1 | 5 | **173,982** | **490,887** | 21 | 8 | 8 |
| 5 | 4 | 4 | **249,607** | 89 | 11 | 6 | 14 |
| 4 | 2 | 3 | **95,118** | 52 | 7 | 7 | 24 |
| 5 | 2 | 6 | **25,638** | 38 | 12 | 8 | 7 |
| 4 | 2 | 3 | **7,470** | 23 | 6 | 4 | 6 |
| 5 | 3 | 3 | **2,453** | 13 | 10 | 6 | 3 |
| 3 | 4 | 4 | **995** | 9 | 5 | 5 | 5 |
| 1.0 | 3.0 | **9059.9** | **567,796.0** | 64.0 | 6.0 | 2.0 | 6.0 |
| 1.0 | 0.0 | **283687.1** | 7.0 | 21.0 | 0.0 | 6.0 | 1.0 |
| 5.0 | 3.0 | **2006639.2** | 6.0 | 1.0 | 4.0 | 2.0 | 1.0 |
| 1.0 | 1.0 | **3530678.4** | 7.0 | 2.0 | 0.0 | 3.0 | 1.0 |
| 1.0 | 7.0 | **1829354.6** | 4.0 | 3.0 | 3.0 | 0.0 | 2.0 |
| 4.0 | 1.0 | **352734.0** | 2.0 | 1.0 | 3.0 | 10.0 | 1.0 |
| 4.0 | 5.0 | **25776.2** | 2.0 | 4.0 | 4.0 | 2.0 | 2.0 |
| 1.0 | 6.0 | **816.0** | 1.0 | 5.0 | 3.0 | 3.0 | 1.0 |
| 2.0 | 3.0 | **8,038,745.5** | 2.0 | 4.0 | 2.0 | 5.0 | 1.0 |
| 6.0 | 10.0 | 40.0 | 1.0 | 8.0 | 2.0 | 3.0 | 3.0 |
| 0.0 | 10.0 | 29.0 | 7.0 | 9.0 | 15.0 | 17.0 | 21.0 |
| 3.0 | 91.0 | 17.0 | 10.0 | 7.0 | 6.0 | 14.0 | 18.0 |
| 2.0 | **1078.0** | 15.0 | 8.0 | 11.0 | 8.0 | 8.0 | 21.0 |

| | | | | | | | |
|---|---|---|---|---|---|---|---|
| 0.0 | **6766.6** | 16.0 | 13.0 | 11.0 | 12.0 | 13.0 | 87.0 |
| 2.0 | **13387.3** | 10.0 | 10.0 | 11.0 | 8.0 | 12.0 | 172.0 |
| 7.0 | **12990.9** | 15.0 | 8.0 | 9.0 | 17.0 | 44.0 | 187.0 |
| 3.0 | **7611.0** | 20.0 | 10.0 | 10.0 | 7.0 | 4.0 | 93.0 |
| 4.0 | **4096.6** | 12.0 | 8.0 | 9.0 | 11.0 | 5.0 | 56.0 |
| 4.0 | **2196.2** | 13.0 | 7.0 | 17.0 | 6.0 | 10.0 | 38.0 |
| 1.0 | **996.0** | 7.0 | 11.0 | 11.0 | 5.0 | 7.0 | 27.0 |
| 38.0 | **49,122.6** | 48.0 | 17.0 | 44.0 | 16.0 | 25.0 | 24.0 |
| **275.0** | 137.0 | 18.0 | 8.0 | 13.0 | 10.0 | 5.0 | 9.0 |
| **2605.2** | 42.0 | 21.0 | 8.0 | 15.0 | 15.0 | 15.0 | 20.0 |
| **12445.4** | 12.0 | 5.0 | 7.0 | 8.0 | 5.0 | 7.0 | 21.0 |
| **24269.8** | 9.0 | 10.0 | 8.0 | 7.0 | 6.0 | 27.0 | 21.0 |
| **29877.2** | 6.0 | 13.0 | 10.0 | 9.0 | 9.0 | 22.0 | 11.0 |
| **16463.7** | 24.0 | 39.0 | 28.0 | 42.0 | 13.0 | 36.0 | 22.0 |
| **19185.1** | 14.0 | 31.0 | 11.0 | 22.0 | 14.0 | 21.0 | 17.0 |
| **17493.7** | 7.0 | 5.0 | 8.0 | 15.0 | 9.0 | 13.0 | 15.0 |
| **13081.0** | 6.0 | 5.0 | 6.0 | 14.0 | 20.0 | 11.0 | 11.0 |
| **6622.5** | 3.0 | 6.0 | 6.0 | 6.0 | 10.0 | 6.0 | 14.0 |
| **6420.5** | 5.0 | 21.0 | 9.0 | 22.0 | 15.0 | 20.0 | 16.0 |
| **4442.7** | 16.0 | 15.0 | 10.0 | 14.0 | 8.0 | 14.0 | 6.0 |
| **2775.3** | 20.0 | 17.0 | 15.0 | 32.0 | 19.0 | 24.0 | 27.0 |
| **2149.2** | 4.0 | 7.0 | 6.0 | 11.0 | 12.0 | 6.0 | 11.0 |
| **1393.1** | 8.0 | 4.0 | 8.0 | 10.0 | 8.0 | 11.0 | 20.0 |
| **159,499.4** | 18.0 | 6.0 | 15.0 | 0.0 | 5.0 | 3.0 | 146.0 |
| 96.0 | 0.0 | 12.0 | 2.0 | 2.0 | 2.0 | 10.0 | 4.0 |
| 96.0 | 6.0 | 194.0 | 54.0 | 142.0 | 692.0 | 84.0 | 6.0 |
| 44.0 | 0.0 | 2.0 | 20.0 | 14.0 | 10.0 | 8.0 | 10.0 |
| 38.0 | 0.0 | 4.0 | 52.0 | 60.0 | 0.0 | 12.0 | 8.0 |
| 24.0 | 0.0 | 4.0 | 144.0 | 148.0 | 12.0 | 12.0 | 6.0 |
| 34.0 | 6.0 | 0.0 | 318.0 | 290.0 | 4.0 | 36.0 | 6.0 |
| 22.0 | 8.0 | 0.0 | 520.0 | 574.0 | 10.0 | 42.0 | 4.0 |
| 26.0 | 4.0 | 2.0 | 810.0 | 840.0 | 12.0 | 68.0 | 2.0 |
| 28.0 | 8.0 | 4.0 | 942.0 | 964.0 | 42.0 | 106.0 | 10.0 |
| 20.0 | 0.0 | 8.0 | 788.0 | 768.0 | 14.0 | 66.0 | 12.0 |
| 16.0 | 4.0 | 2.0 | 816.0 | 746.0 | 32.0 | 70.0 | 6.0 |
| 104.0 | 4.0 | 10.0 | 200.0 | 232.0 | 18.0 | 56.0 | 14.0 |
| 12.0 | 0.0 | 4.0 | 286.0 | 232.0 | 4.0 | 28.0 | 8.0 |
| 14.0 | 2.0 | 2.0 | 92.0 | 116.0 | 4.0 | 16.0 | 8.0 |
| 22.0 | 6.0 | 2.0 | 22.0 | 48.0 | 2.0 | 10.0 | 4.0 |
| 4.0 | 4.0 | 2.0 | 8.0 | 12.0 | 6.0 | 6.0 | 6.0 |
| 6.0 | 6.0 | 6.0 | 8.0 | 6.0 | 4.0 | 2.0 | 6.0 |
| 33 | 3 | 24 | 70 | 14 | 8 | 9 | 6 |
| 18 | 8 | 3967 | 152 | 28 | 1902 | 8 | 9 |
| 19 | 10 | 23035 | 518 | 154 | 10867 | 1 | 5 |
| 13 | 68 | 12583 | 278 | 91 | 6170 | 2 | 0 |
| 6 | 1008 | 868 | 20 | 4 | 8 | 6 | 2 |
| 2.0 | 11052.3 | 46.0 | 4.0 | 2.0 | 2.0 | 6.0 | 8.0 |
| 12.0 | 60025.8 | 152.0 | 4.0 | 4.0 | 8.0 | 4.0 | 6.0 |

| | | | | | | | |
|---|---|---|---|---|---|---|---|
| 8.0 | 6867.7 | 8.0 | 2.0 | 0.0 | 0.0 | 10.0 | 4.0 |
| 12.0 | 160.0 | 34.0 | 4.0 | 4.0 | 6.0 | 10.0 | 6.0 |
| 14.0 | 308.0 | 52.0 | 4.0 | 8.0 | 10.0 | 6.0 | 10.0 |
| 10.0 | 574.0 | 76.0 | 2.0 | 4.0 | 6.0 | 2.0 | 14.0 |
| 6.0 | 226.0 | 32.0 | 2.0 | 0.0 | 2.0 | 2.0 | 2.0 |
| 8.0 | 4.0 | 4.0 | 4.0 | 2.0 | 4.0 | 2.0 | 2.0 |
| 7 | 14 | 1 | 5 | 3 | 5 | 0 | 6 |
| 4 | 59 | 4 | 6 | 7 | 6 | 1 | 1 |
| 7 | 78 | 0 | 4 | 1 | 0 | -1 | 4 |
| 11 | 24 | 6 | 6 | 7 | 5 | 3 | 3 |
| 7 | 5 | 6 | 7 | 0 | 2 | -1 | 2 |
| 6 | 6 | 4 | 7 | 5 | 3 | 2 | 3 |

| Lu 175 (cps) |
|---|
| 2.0 |
| 3.0 |
| **1048.0** |
| **84445.9** |
| **999016.6** |
| **1832911.4** |
| **743822.2** |
| **79858.6** |
| **5880.2** |
| **5672.1** |
| **3813.5** |
| **3094.3** |
| **449.0** |
| **3,759,562.866** |
| |
| 3.0 |
| 0.0 |
| 4.0 |
| 39 |
| 16 |
| 16 |
| 9 |
| 9 |
| 6 |
| 6 |
| 3 |
| 6 |
| 6 |
| 5 |
| 9 |
| 121.0 |
| 3319.4 |
| 20003.0 |
| 36001.3 |
| 17843.1 |
| 3886.5 |
| 262.0 |
| 15.0 |
| 2.0 |
| 4.0 |
| 11.0 |
| 5.0 |
| 4.0 |

| |
|---|
| 12.0 |
| 19.0 |
| 30.0 |
| 12.0 |
| 17.0 |
| 6.0 |
| 11.0 |
| 42.0 |
| 12.0 |
| 14.0 |
| 9.0 |
| 6.0 |
| 11.0 |
| 26.0 |
| 9.0 |
| 9.0 |
| 5.0 |
| 13.0 |
| 14.0 |
| 17.0 |
| 47.0 |
| 18.0 |
| 28.0 |
| 131084.7 |
| 2.0 |
| 10.0 |
| 0.0 |
| 0.0 |
| 0.0 |
| 4.0 |
| 2.0 |
| 4.0 |
| 6.0 |
| 0.0 |
| 8.0 |
| 0.0 |
| 4.0 |
| 6.0 |
| 0.0 |
| 0.0 |
| 0.0 |
| 1 |
| 3 |
| 2 |
| 0 |
| 4 |
| 0.0 |
| 2.0 |

2.0
2.0
0.0
2.0
0.0
6.0
-1
2
1
0
0
0

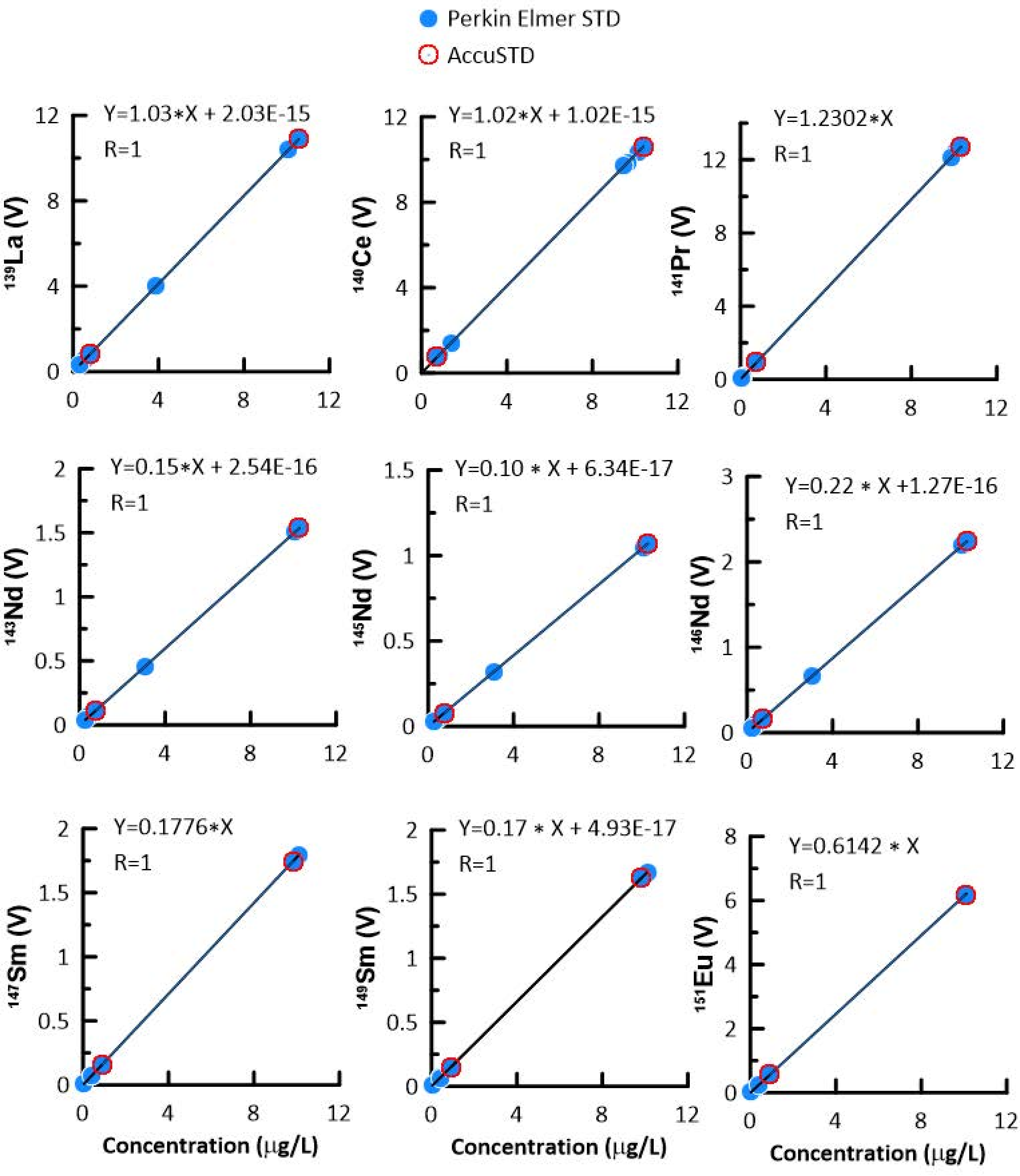


Supplementay Fig. 1. Calibration curve between Ion beam intensity (V) and concentration (mg/L) of each REE isotope

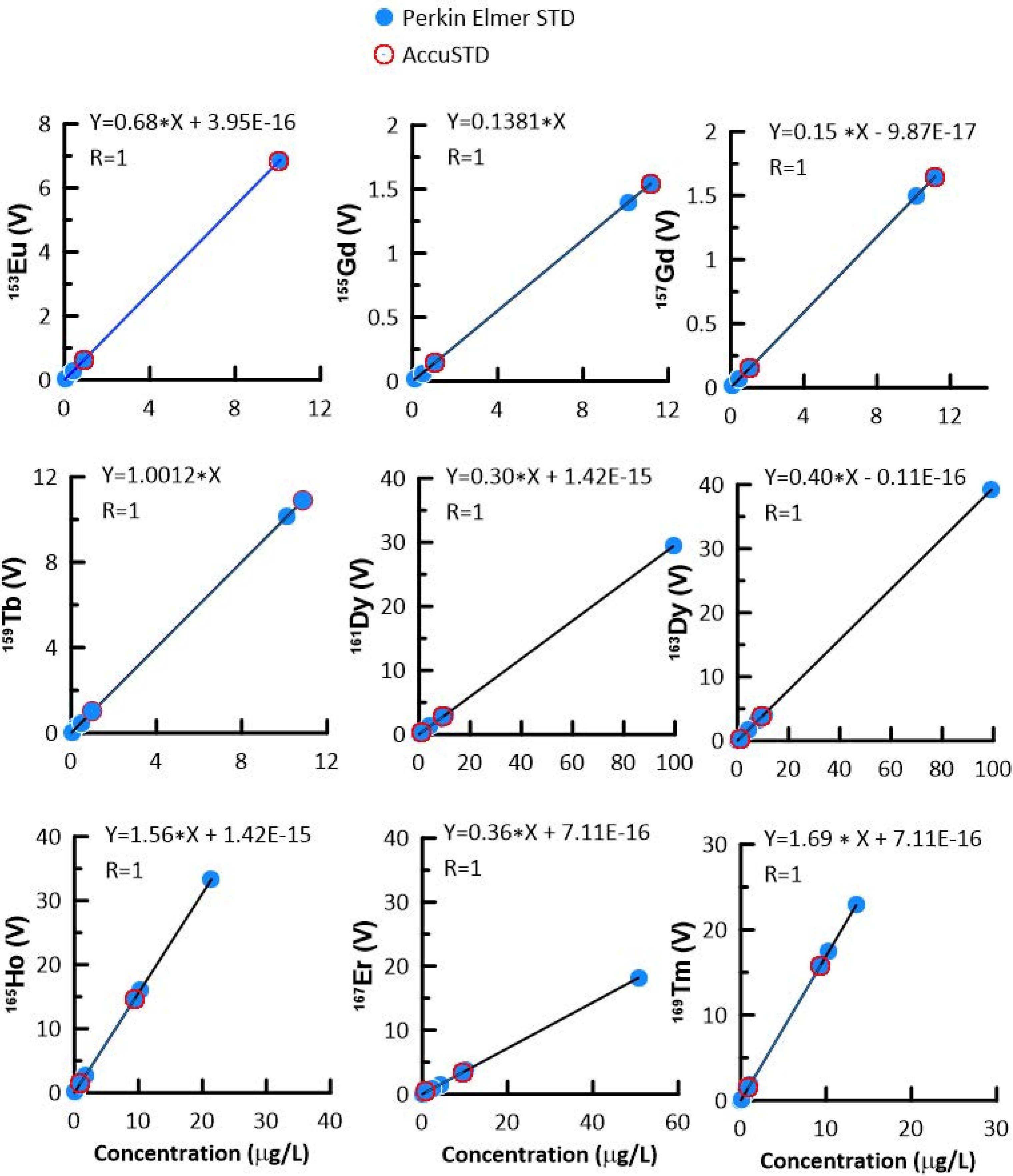


Supplementay Fig. 1. continued.

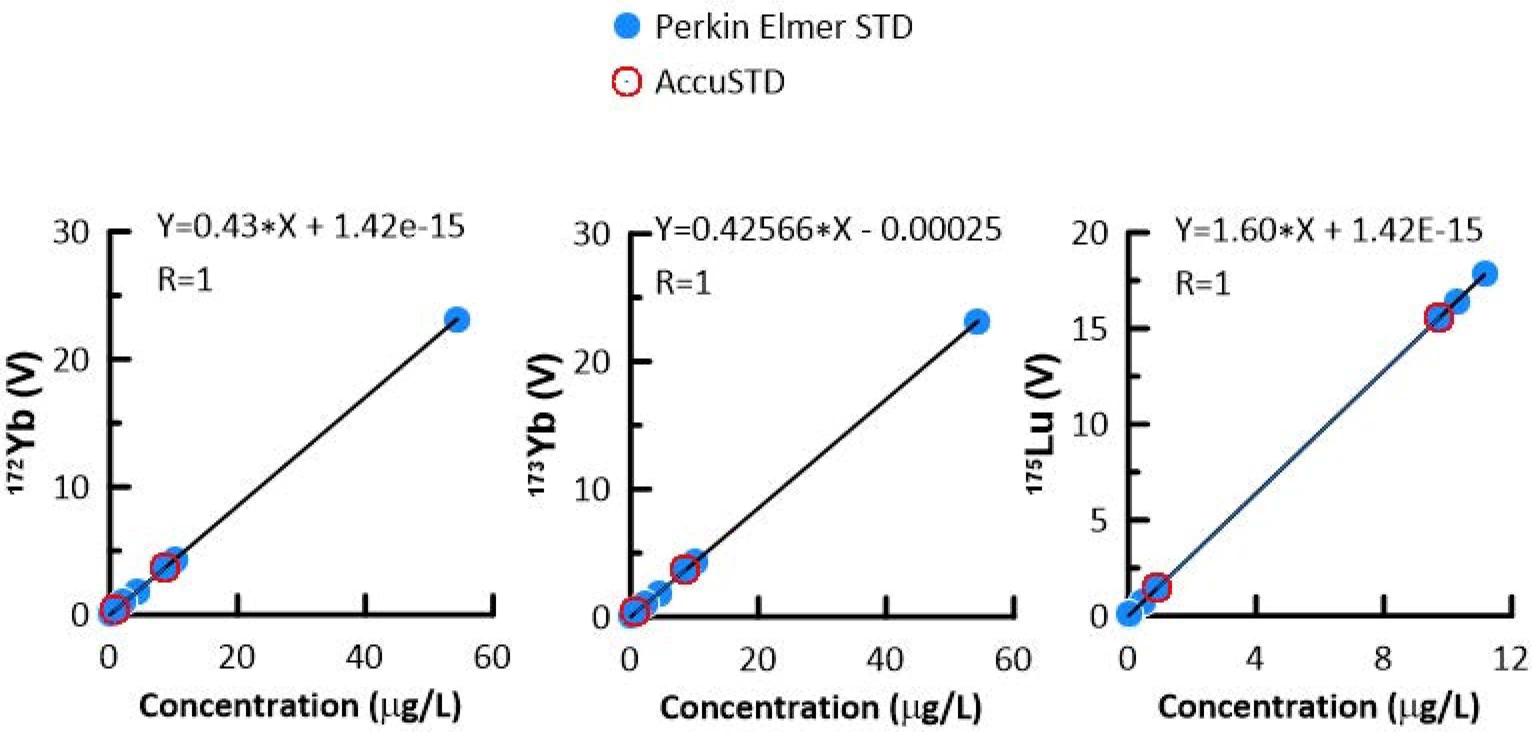


Supplementay Fig. 1.
